# SlBIR3 Negatively Regulates PAMP Responses and Cell Death in Tomato

**DOI:** 10.3390/ijms18091966

**Published:** 2017-09-13

**Authors:** Shuhua Huang, Shuming Nie, Shufen Wang, Jianwei Liu, Yanfeng Zhang, Xiaofeng Wang

**Affiliations:** 1 State Key Laboratory of Crop Stress Biology in Arid Areas, College of Horticulture, Northwest A&F University, Yangling 712100, China; hsh813@126.com (S.H.); nieshuming@163.com (S.N.); sfwang0321@163.com (S.W.); yuanshangty@163.com (J.L.); zhangyfcl@126.com (Y.Z.); 2 Hybrid Rapeseed Research Center of Shanxi Province, Yangling 712100, China

**Keywords:** tomato, *SlBIR3*, BR signaling, PTI responses, *Botrytis cinerea*, cell death

## Abstract

Bri1-associated kinase 1 (BAK1)-interacting receptor-like kinase (BIR) proteins have been shown to play important roles in regulating growth and development, pathogen associated molecular pattern (PAMP)-triggered immunity (PTI) responses, and cell death in the model plant, *Arabidopsis thaliana*. We identified four BIR family members in tomato (*Solanum lycopersicum*), including *SlBIR3*, an ortholog of *AtBIR3* from *A. thaliana*. *SlBIR3* is predicted to encode a membrane localized non-arginine-aspartate (non-RD) kinase that, based on protein sequence, does not have autophosphorylation activity but that can be phosphorylated in vivo. We established that SlBIR3 interacts with SlBAK1 and AtBAK1 using yeast two-hybrid assays and co-immunoprecipitation and maltose-binding protein pull down assays. We observed that *SlBIR3* overexpression in tomato (cv. micro-tom) and *A. thaliana* has weak effect on growth and development through brassinosteroid (BR) signaling. *SlBIR3* overexpression in *A. thaliana* suppressed flg22-induced defense responses, but did not affect infection with the bacterial pathogen *Pseudomonas syringae* (*Pst*DC3000). This result was confirmed using virus-induced gene silencing (VIGS) in tomato in conjunction with *Pst*DC3000 infection. Overexpression of *SlBIR3* in tomato (cv. micro-tom) and *A. thaliana* resulted in enhanced susceptibility to the necrotrophic fungus *Botrytis cinerea*. In addition, co-silencing *SlBIR3* with *SlSERK3A* or *SlSERK3B* using VIGS and the tobacco rattle virus (TRV)-RNA2 vector containing fragments of both the *SlSERK3* and *SlBIR3* genes induced spontaneous cell death, indicating a cooperation between the two proteins in this process. In conclusion, our study revealed that *SlBIR3* is the ortholog of *AtBIR3* and that it participates in BR, PTI, and cell death signaling pathways.

## 1. Introduction

Plants are often subjected to various adverse environmental conditions during their entire life cycle, which are detrimental to growth and development [1,2,3,4]. Consequently, they have evolved various strategies to perceive and rapidly adapt to external environmental conditions. Response mechanisms include leucine-rich repeats receptor-like protein kinases (LRR-RLKs), the largest subgroup of the receptor-like protein kinase (RLKs) family in plants, which play vital roles in transmitting extracellular environmental stimuli to intracellular chemical signals [5,6,7]. Typical LRR-RLKs are composed of an extracellular leucine-rich repeat domain that senses a specific external environmental stimulus, a single-pass transmembrane domain that localizes the protein to the plasma membrane and an intracellular domain that relays the downstream intracellular signal via a sequential phosphorylation mechanism [8,9,10]. Based on the presence of an arginine (R) residue preceding the conserved catalytic aspartic acid (D) residue in subdomain VIb of the cytoplasmic kinase domain, the LRR-RLK proteins are classified as RD kinases and non-RD kinases. RD kinases, which can be activated through the autophosphorylation of their activation loop, have this R residue, while non-RD kinases do not [11,12,13]. The activation of the non-RD kinases usually depends on their specific substrates, which differ from those of the RD kinases [14]. Notably, the non-RD kinases are usually associated with plant innate immunity signaling [15].

At present, the most extensively studied RD kinases are the receptors BRs, Brassinosteroid-insensitive 1 (BRI1) and BAK1, which play essential roles in brassinosteroid (BR) signaling [16,17,18]. BR interacts with a 94-amino-acid binding domain, composed of an island domain, a 70 amino acid region between the 21st and 22nd LRR, and a C-terminal flanking motif, comprising a 24-amino-acid LRR22, in the ectodomain of BRI1, thereby inducing its autoactivation [19]. The activated BRI1 then phosphorylates its substrate, BKI1 (BRI1 kinase inhibitor 1) [20], a negative regulator of BR signaling, at Y211, and causes its rapid dissociation from the cell surface [21], which allows the formation of a BRI1 and BAK1 heterodimer [16,17,18]. After recruitment of BAK1 by the activated BRI1 to form a heterodimer, the threonine residues T446, T449, T450 and T455 in the active loop of BAK1 are phosphorylated by BRI1, resulting in the activation of BAK1. Activated BAK1 fully trans-activates BRI1 by phosphorylating sites in its juxtamembrane region (S838, T846, S858) and C-terminal region (S1166, T1180) [9]. The cytoplasmic receptor–like kinases, BR-Signaling kinase 1/2 (BSK1/2) and constitutive differential growth 1 (CDG1), are then phosphorylated by the fully activated BRI1 and released from the BRI1-BSKs complex [22,23]. The activated BSKs interact and activate a downstream substrate phosphatase, *BRI1*-suppressor 1 (BSU1), which further associates with and dephosphorylates the phosphorylated brassinosteroid insensitive 2 (BIN2), inhibiting its activity [24]. Finally, dephosphorylated BIN2 promotes the accumulation of dephosphorylated brassinazole resistant 2 (BZR2)/*BRI1*-ems suppressor 1 (BES1) in the nucleus, which can promote the expression of BR-responsive genes [25] to regulate growth and development.

Processes known to be affected by this signaling mechanism include seed formation and germination, root hair development, cell division, expansion and differentiation, shoot branching, accelerated flowering, pollen and anther development, photomorphogenesis, senescence and enhanced resistance to diseases and stresses [26]. The well-studied non-RD kinase receptor flagellinsensing 2 (FLS2), is the flagellin receptor, which plays a central role in PAMP-triggered plant immunity (PTI) responses [27]. During PTI signaling, when flagellin is perceived by FLS2, a flagellin-dependent association between FLS2 and BAK1 occurs, followed by the rapid phosphorylation of *Botrytis*-induced kinase 1 (BIK1). The activated BIK1 protein phosphorylates BAK1 and FLS2, which may also transphosphorylate BIK1, and potentially some other downstream substrates, causing the dissociation of the activated BIK1 from the plasma membrane into the protoplast to initiate immune signaling [28]. Once the immune signaling system is activated, negative regulation of this kinase is essential to protect the plant from excessive damage. Plant u-box 12 (PUB12) and Plant u-box 13 (PUB13), two U-box type E3 ubiquitin ligases, are phosphorylated by BAK1 during flagellin-induced signaling, followed by the association with FLS2. This precedes the ubiquitination of FLS2 by PUB12 and PUB13 and results in FLS2 degradation [29].

Based on the studies described above, we conclude that BAK1 may be an adaptor of multiple membrane-localized receptors with distinct functions but a common RD-type regulatory activity involving several RD and non-RD kinases in the control of growth and innate immunity. BAK1, also named somatic embryogenesis-related kinase 3 (SERK3) for its role in regulating somatic development [17,18], has four plant paralogs in *A. thaliana* with different functions [30]. Somatic embryogenesis-related kinase 1 (SERK1) and somatic embryogenesis-related kinase 2 (SERK2) regulate anther development [31,32] and all the paralogs show functional redundancy when mediating BR signaling [33]. SERK3 and somatic embryogenesis-related kinase 4 (SERK4) act together to regulate cell death in a BR independent manner [34,35,36,37], and studies also suggest that the BAK1 interacting proteins, BIR1 and BIR2 can suppress cell death and defense responses [38,39,40]. The BIR1 null mutant was reported to exhibit a spontaneous cell death phenotype and constitutive activation of defense responses, which can be rescued by the functional loss of suppressor of bak1-interacting receptor like kinase 1 (BIR1)-1 (SOBIR1), calreticulin 3 (CRT3), ER-localized DnaJ-like protein 3b (ERdj3b), stromal-derived factor-2 (SDF2), or BAK1 [35,39,41]. BIR2, an atypical non-RD LRR-RLK, constitutively associates with BAK1 and interferes with the interaction between BAK1 and FLS2, resulting in the negative regulation of immune signaling and cell death, but has no effect on BR signaling [38].

Studies to date have focused particularly on the role of BIR1 and BIR2 in PTI signaling, resistance to fungal infection, control of cell death in association with, BAK1, but less is known about the involvement of BIR3 proteins. Here, a tomato ortholog to *A. thaliana AtBIR3* (*At*1g27190), *SlBIR3* (*Solyc*05g006570.1.1), was cloned and characterized. Genetic data suggested that SlBIR3 has weak effect on BR signaling and suppress PAMP responses. We verified that a developed Agrobacterium-mediated tobacco rattle virus (TRV)-based virus-induced gene silencing (VIGS) method [35,42] with TRV-RNA2 vector containing fragments of both the *SlSERK3* and *SlBIR3* genes was reliable and effective in tomato, and used this approach to show that SlBIR3 together with SlSERK3 suppresses cell death.

## 2. Results

### 2.1. SlBIR3 Encodes a Membrane Localized Atypical Receptor-Like Protein Kinase

To clone the ortholog of *A. thaliana AtBIR3* from tomato, a basic local alignment search tool (BLAST) search was conducted amongst the protein sequences released with the tomato genome (ITAG release 3.10) database (https://solgenomics.net/tools/blast/), using the AtBIR3 amino acid sequence as a query. The three best-matching related genes were Solyc05g006570.1.1, Solyc02g067560.1.1, and Solyc02g087460.1.1, all of which had an e-value of (<1 × 10^−180^) Similar results were obtained when the AtBIR2 (*At*3g28450) or AtBIR4 (*At*1g69990) amino acid sequences were used as a query. An amino acid sequence alignment between these three proteins and AtBIR3 showed that Solyc05g006570.1.1, Solyc02g067560.1.1 and Solyc02g087460.1.1 encode proteins with 64%, 61% and 60% identity with AtBIR3 (*At*1g27190), (Figure S1), respectively, indicating that Solyc05g006570.1.1 may be the tomato ortholog of AtBIR3.

To confirm this, a phylogenetic analysis was carried out, in which orthologous genes from other species were identified from a plant membrane protein database (http://aramemnon.uni-koeln.de/). Phylogenetic analysis showed Solyc02g067560.1.1 and Solyc02g087460.1.1 clustered together with AtBIR2, and Solyc05g006570.1.1 aligned within the same clade as AtBIR3 and AtBIR4 (Figure S2), suggesting that the closest ortholog of AtBIR3 in tomato is Solyc05g006570.1.1, which we renamed SlBIR3.

**Figure 1:**
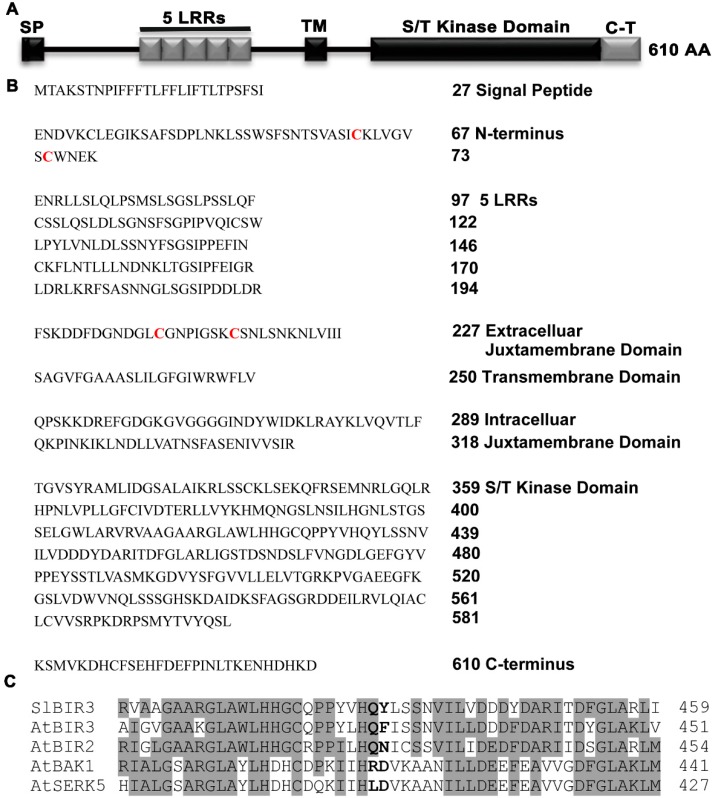
*SlBIR3* encodes a leucine rich repeat (LRR) receptor-like protein kinase. (**A**) Domain structure of *SlBIR3*. SP, signal peptide: LRR, leucine-rich repeat; TM, transmembrane domain; S/T kinase domain, serine/threonine kinase domain; C-T, carboxy-terminal. (**B**) *SlBIR3* encodes an LRR receptor like kinase (RLK). The numbers indicate the corresponding amino acid position relative to the initiation methionine (M). In the extracellular domain, the structural backbone contains a signal peptide, an N-terminus with the conserved cysteine pair residues highlighted in red, 5 LRRs, and one extracellular juxtamembrane domain with the conserved cysteine pair residues highlighted in red; in the intracellular domain, the structural backbone contains an intracellular juxtamembrane domain, a cytoplasmic domain and a carboxy-terminal domain. The intra- and extra-cellular domains are separated by a transmembrane domain. (**C**) Alignment of partial kinase domain sequences of SlBIR3, AtBIR3, AtBIR2, AtBAK1 and AtSERK5. The identical amino acids are shaded gray and the RD motif are highlighted in bold.

An analysis of the deduced SlBIR3 protein sequence indicated that it contains an N-terminal signal peptide, five LRR regions, an extracellular juxtamembrane domain, a transmembrane domain, an intracellular juxtamembrane domain, a Ser/Thr cytoplasmic domain and a carboxy-terminal domain (Figure 1A,B). These structures are almost identical to those of AtBIR2 and AtBIR3. A sequence alignment of the predicted kinase domain of SlBIR3, AtBIR3, AtBIR2, AtBAK1 and somatic embryogenesis-related kinase 5 (AtSERK5) revealed that SlBIR3 lacks a highly conserved R and the conserved catalytic D residue in subdomain VIb of the kinase domain (Figure 1C). This is important for the catalytic function and kinase activity and so we concluded that SlBIR3 is a non-RD kinase, and that SlBIR3 is an atypical membrane localized receptor-like protein kinase.

**Figure 2:**
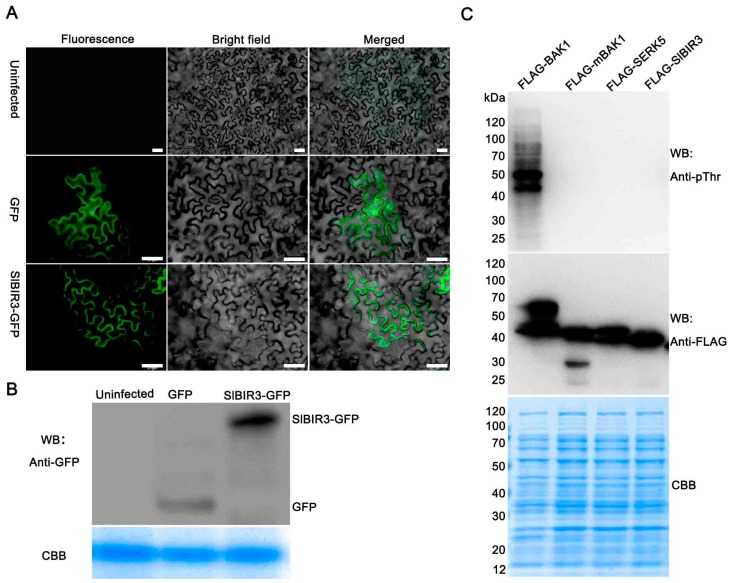
Subcellular localization and phosphorylation activity of SlBIR3. (**A**) Subcellular localization of SlBIR3-GFP (green fluorescent protein). Agrobacterium-mediated transient transformation of tobacco epidermal cells by infiltration. Left panels show green fluorescence signal under blue light. Middle panels show the bright field images. The right panels show the merged images. The lower panels show that the SlBIR3-GFP fusion protein mainly localizes to the plasma membrane. The top panels show that the non-infected tobacco leaf cells do not have green fluorescence signal and the middle panels show that the GFP is distributed throughout the control cells. Scale bars, 50 μm. (**B**) Western blot analysis of the expression of SlBIR3-GFP shown in (**A**) detected using an anti-GFP antibody. Coomassie brilliant blue (CBB) staining shows equal protein loading. (**C**) In vitro SlBIR3 phosphorylation activity. The phosphorylation activity of the recombinant FLAG-SlBIR3, FLAG-BAK1, FLAG-mBAK1 (L317E) and FLAG-SERK5 proteins was detected using an anti-pThr antibody, and aliquots of the recombinant proteins were detected in different gels using anti-FLAG antibodies in Western blot analysis. CBB staining (bottom) shows equal loading.

To further confirm that SlBIR3 is a membrane localized receptor-like kinase, a subcellular localization experiment was performed, using agrobacterium mediated transient transformation of tobacco epidermal cells. The results suggested that SlBIR3 is mainly localized to the plasma membrane (Figure 2A), which is the same localization as that previously reported for BAK1 [18]. An in vitro phosphorylation assay was also performed to test whether SlBIR3 has autophosphorylation and transphosphorylation activities. As shown in Figure 2C, only the recombinant FLAG-BAK1 protein showed strong autophosphorylation of the threonine residues and also had the ability to transphosphorylate *E. coli* proteins on these. Neither autophosphorylation of recombinant FLAG-SlBIR3 nor the phosphorylation of *E. coli* proteins transphosphorylated by FLAG-SlBIR3 on the threonine residues was detected by the anti-pThr antibody, similar to the inactive recombinant kinase proteins FLAG-mBAK1(317E) and FLAG-AtSERK5. These results indicated that SlBIR3 is a membrane localized atypical receptor-like protein kinase.

### 2.2. SlBIR3 Interacts with SlBAK1 and AtBAK1

**Figure 3:**
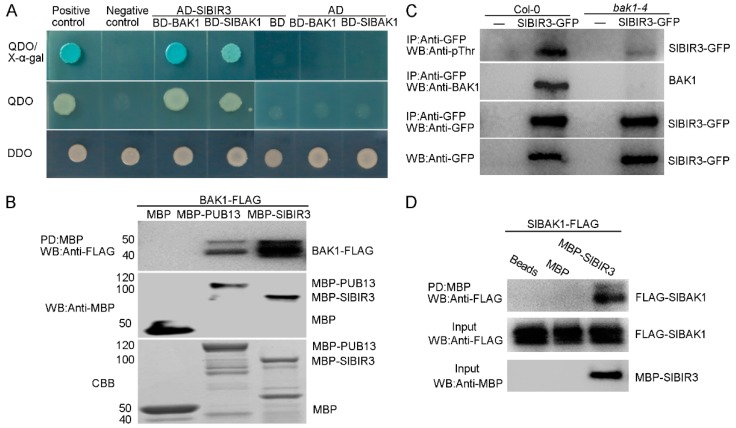
SlBIR3 interacts with BAK1 and SlBAK1. (**A**) SlBIR3 interacts with BAK1 and SlBAK1 in yeast. Yeast strain AH109 containing the BAK1 cytoplasmic domain in the prey vector pGADT7 and BAK1 or SlBAK1 cytoplasmic domain in bait vector pGBKT7 were tested for growth on SD/-Leu-Trp (DDO), SD/-Leu-Trp-His-Ade (QDO), and SD/-Leu-Trp-His-Ade+X-a-gal (QDO/X-α-gal). The positive control was AH109:pGBKT7-53/pGADT7-RecT, the negative control were AH109:pGBKT7-Lam/pGADT7-RecT, AH109:pGBKT7/pGADT7-SlBIR3, AH109:pGBKT7-BAK1/pGADT7, and AH109:pGBKT7-SlBAK1/pGADT7; (**B**) SlBIR3 interacts with BAK1 in vitro. A MBP pull down assay was performed by incubating FLAG-tagged BAK1 cytoplasmic domain with MBP-tagged SlBIR3 cytoplasmic domain. The MBP-bound beads were used as a negative control and the MBP-tagged PUB13 cytoplasmic domain was used as a positive control. Bound proteins were subjected to Western blot analysis with an anti-FLAG antibody. The input proteins were analyzed using an anti-MBP antibody. CBB staining shows the protein inputs; (**C**) SlBIR3 interacts with BAK1 in vivo. The total proteins extracted from 11-day-old *A. thaliana* expressing SlBIR3-GFP in the Col-0 or *bak1-4* background were immunoprecipitated with anti-GFP antibody and protein A agarose beads. The total and immunoprecipitated proteins were analyzed with anti-GFP, anti-pThr, and anti-BAK1 antibodies; (**D**) SlBIR3 interacts with SlBAK1 in vitro. A MBP pull down assay was performed by incubating FLAG-tagged SlBAK1 cytoplasmic domain with MBP-tagged SlBIR3 cytoplasmic domain. MBP-bound beads and MBP protein were used as a negative control. The bound proteins were subjected to Western blot analysis with an anti-FLAG antibody. The input proteins were analyzed with an anti-MBP antibody.

Previous studies have indicated that AtBIR3 interacts with AtBAK1 [38]. Since SlBIR3 is the tomato ortholog of AtBIR3, we investigated whether SlBIR3 could interact with SlBAK1 (SlSERK3B) and AtBAK1. An activation domain (AD) fused to the cytoplasmic domain of SlBIR3 (AD-SlBIR3) and a DNA-binding domain (BD) fused to the kinase domain of SlBAK1 and AtBAK1 (BD-SlBAK1, BD-BAK1) were generated for expression in yeast. As shown in Figure 3A, separate co-expression of AD-SlBIR3 with BD-SlBAK1 and BD-BAK1, conferred yeast cell growth on the SD/-Trp-Leu-Ade-His selection medium, as well as X-α-galactosidase activity. This demonstrated that SlBIR3 interacts with SlBAK1 and AtBAK1 in yeast.

Next, a pull-down assay was carried out to verify the interaction between SlBIR3 protein and BAK1. The cytoplasmic domain of SlBIR3 fused to maltose binding protein (MBP) immobilized on amylose resin was used as a bait to bind BAK1-FLAG fusion proteins. As shown in Figure 3B, BAK1-FLAG interacted with MBP-SlBIR3, the BAK1-FLAG and MBP-PUB13 (positive control), but not with MBP alone, indicating that SlBIR3 physically interacts with BAK1 in vitro.

We then performed a co-immunoprecipitation analysis to test the interaction between SlBIR3 and BAK1 in vivo. First, a construct expressing SlBIR3-GFP protein driven by the CaMV 35S promoter was transformed into *Arabidopsis thaliana* Col-0 and the *A. thaliana bak1-4* mutant. The transgenic plants were grown in liquid culture for 11 days and then protein extracts were made and subjected to immunoprecipitation using anti-GFP antibody and protein A agarose beads. The immunoprecipitated proteins were subjected to Western blot analysis with an anti-BAK1 antibody. As shown in Figure 3C, BAK1 was detected in the immunoprecipitated proteins from total protein extracts of SlBIR3 transgenic plants in Col-0, but not in extracts from wild type (Col-0), *bak1-4* or *SlBIR3* overexpression lines in the *bak1-4* background. We noted that the phosphorylation of SlBIR3-GFP detected with an anti-pThr antibody in wild type was much stronger than that in *bak1-4*, indicating *Sl*BIR3 may be phosphorylated by BAK1 *in planta*, and that SlBIR3 is a substrate of BAK1.

To further characterize the association between SlBIR3 and SlBAK1, another MBP pull-down assay was performed using MBP-tagged SlBIR3 and FLAG-tagged SlBAK1 cytoplasmic domain expressed in *E. coli*, which were purified using FLAG agarose beads and MBP amylose resin, respectively. As shown in Figure 3D, the SlBAK1-FLAG proteins interacted with MBP-SlBIR3 but not with amylose resin (beads) or MBP. Taken together, these results were consistent with an association of SlBIR3 with BAK1 and SlBAK1.

### 2.3. Overexpressing SlBIR3 Has Weak Effect on BR Signaling

Previous results indicated that BAK1 interacts with BRI1 to initiate early steps in BR signaling, resulting in the promotion of growth and development [9,17,18]. Since SlBIR3 interacts with BAK1, we reasoned that *SlBIR3* may be involved in regulating growth and developmental processes through BR signaling. To test this hypothesis, a construct expressing the SlBIR3-GFP fusion protein driven by CaMV35S promoter was introduced into the micro-tom tomato cultivar. As shown in Figure S3, transgenic lines were obtained and expression of the transgene and recombinant protein verified by semi RT-PCR and Western blot analysis, respectively. We observed that overexpression of *SlBIR3-GFP* had no significant effect on growth or developmental features though appeared a slight reduction on plant height and expansion diameter, and no significant changes were detected in the transcript levels of the BR-responses genes *SlCPD* and *SlDWF4* between SlBIR3 transgenic lines and wild type plants. These results suggested that *SlBIR3* overexpression in tomato has weak effect on BR signaling.

Next, we tested whether overexpression of *SlBIR3* in *A. thaliana* affects growth and development through BR signaling. The same transgenic construct used for tomato transformation described above was introduced into *A. thaliana* Col-0 by agroinfiltration and several transgenic lines with different recombinant protein expression levels were identified and analyzed. As shown in Figure S4, no significant differences were observed in the phenotypes of the transgenic lines and wild type plants, regardless of the level of SlBIR3-GFP protein accumulation. To further determine whether the BR sensitivity of these transgenic lines was abnormal, a root growth inhibition assay and a hypocotyl elongation assay were carried out. As shown in Figures S5A–E, no significant phenotypic differences were observed as a consequence of overexpressing *SlBIR3* in wild type, but the growth of roots and hypocotyls of *SlBIR3-GFP* overexpression lines were less sensitive to exogenous 24-epiBL in a series of concentrations from 1 to 1000 nM compared to wild type plants. We then determined, using semi RT-PCR, that the expression levels of the BR-responsive genes *constitutive photomorphogenesis and dwarfism* (*CPD*) and *DWARF4* (*DWF4*) were similar in wild type and in *SlBIR3* overexpression seedlings (Figure S5F). These results suggest that SlBIR3 does not dramatically but weakly affect the BR signaling pathway or BR-mediated growth and development in wild-type *A. thaliana*.

### 2.4. SlBIR3 Negatively Regulates PAMP-Triggered Responses but Not Responses to PstDC3000 Treatment

**Figure 4:**
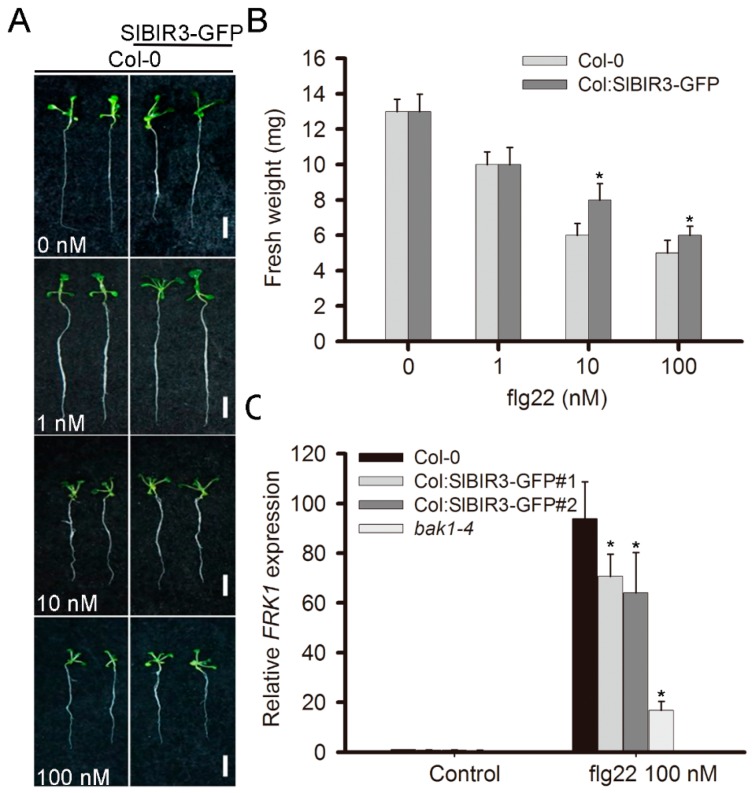
SlBIR3 negative regulates flg22 triggered PAMP responses. (**A**) Phenotypes of Col-0 and SlBIR3-overexpression seedlings (Col-0 background) after flg22 treatment. The photographs show 14-day-old seedlings that were treated with different concentrations of flg22 for 7 days. Scale bars, 10 mm; (**B**) Seedling growth inhibition induced by flg22 is shown in (**A**). At least 12 seedlings were measured; (**C**) The relative expression level of *FRK1* was measured by real time quantitative RT-PCR, with *ACTIN2* used as the reference gene. 14-day-old seedlings were treated with 100 nM flg22 for three hours. Data indicate mean values ± SD and the asterisks indicate significant differences compared to wild-type Col-0, according to a Student’s *t* test (* *p* < 0.05). Experiments were performed three times with similar results.

According to the results of previous studies indicating that BAK1 plays important functions in PTI signaling [27], we speculated that SlBIR3 may also play a role in PAMP-triggered responses. To test this, and to elucidate the biological significance of SlBIR3 in PTI signaling, we tested for seedling growth inhibition by flg22 (a conserved 22 amino acids peptide of flagellin) [43]. *SlBIR3* overexpression lines (Col-0 background) were less sensitive to exogenous flg22 compared to wild type (Col-0) over a range of flg22 concentrations from 10 to 100 nM (Figure 4A,B). It was reported that *FRK1*, an LRR-RLK, is involved in defense responses and was used as a marker gene in early PTI signaling activated by flg22 [44]. Accordingly, we measured the transcript levels of *FRK1* using real-time qRT-PCR. Compared with wild type (Col-0), the expression level of *FRK1* was significantly lower in 14-day-old *SlBIR3* overexpression seedlings (Col-0) after treatment with 100 nM flg22 for 3 h. Meanwhile, we observed that the relative expression level of *FRK1* in *bak1-4* plants was even lower compared to that in wild type (Figure 4C).

We next investigated the role of *SlBIR3* in resistance against plant pathogen *Pseudomonas syringae* pv. *tomato* DC3000 (*Pst*DC3000). Functional analyses of *SlBIR3* were carried out in *SlBIR3* silenced tomato plants (cv. Moneymaker) using a virus-induced gene silencing method. The pTRV2:00 and pTRV2-*SlPDS* vectors were used as negative (VIGS-vector) and positive (VIGS-*SlPDS*) controls, respectively. As previously reported [45], approximately four weeks after infiltration, a clear photo-bleaching phenotype was observed in almost all the positive control plants (Figure S6A). The efficiency of *SlBIR3* silencing in the transgenic plants (VIGS-*SlBIR3*) was assessed by semi-quantitative RT-PCR, which showed that the *SlBIR3* transcript levels were lower than that in negative control (Figure S6B). *Pst*DC3000 infection assays were carried out in VIGS-*SlBIR3* plants and the bacterial growth in VIGS-*Vector* and VIGS-*SlBIR3* groups were very similar to each other (Figure S6C). To further examine the role of SlBIR3 in *Pst*DC3000 resistance, bacterial suspensions were injected into *bak1-4*, Col-0 and *SlBIR3* overexpression lines (all Col-0 background). Bacterial growth in the various genotypes was measured after 4 days post-infection, and no significant differences were observed (Figure S6D).

### 2.5. SlBIR3 Negatively Regulates the Resistance to Botrytis Cinerea in Tomato and A. thaliana

**Figure 5:**
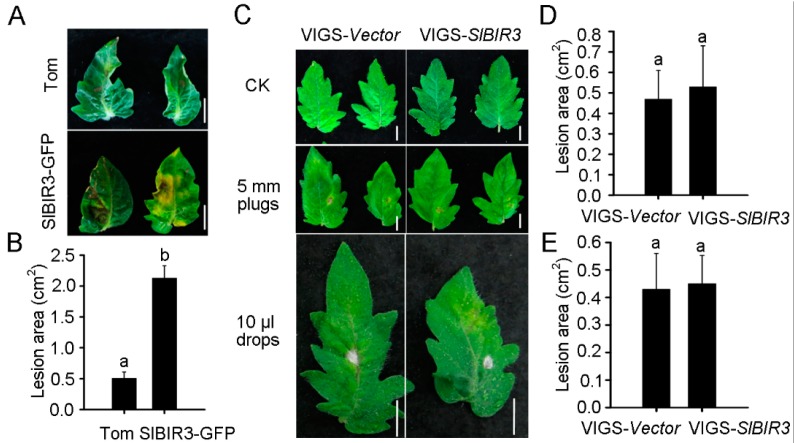
*SlBIR3* suppresses resistance to *B. cinerea* in tomato. (**A**) Disease phenotypes of wild type (Micro-Tom) and SlBIR3-overexpression seedlings (Micro-Tom background) after drop inoculation with 10 μL of *B. cinerea* spores suspension (5 × 10^5^ spores/mL). The photographs show leaves 7 days after the inoculation. The upper panel shows wild type leaves (labeled Tom) and the lower panel shows leaves of SlBIR3-overexpression plants. Scale bars, 10 mm; (**B**) The lesion areas in the leaves shown in (**A**); (**C**) Disease phenotypes of *SlBIR3* silenced leaves (cv. moneymaker). The left panel shows the empty vector control leaves (VIGS-*vector*) and the right panel shows *SlBIR3* silenced leaves (VIGS-*SlBIR3*). The upper panel shows the untreated leaves, the middle panel shows the leaves incubated with 5 mm diameter mycelium plugs of *B. cinerea* and the lower panel shows the leaves incubated with 10 μL drops of a *B. cinerea* spores suspension (5 × 10^5^ spores/mL). Scale bars, 10 mm; (**D**) The lesion areas of leaves incubated with 5 mm diameter mycelium plugs of *B. cinerea* shown in (**C**); (**E**) The lesion areas of leaves incubated with 10 μL drops of *B. cinerea* spores suspension shown in (**C**); The lesion areas of 5 leaves were measured and the values indicate mean values ± SD. Significant differences are indicated by different letters according to a Student’s *t* test (*p* < 0.05). Experiments were performed three times with similar results.

BAK1 has been shown to promote resistance to *Botrytis cinerea* [46]. To investigate whether *SlBIR3* plays a similar role in tomato, detached leaves from wild type (micro-tom background) and *SlBIR3-GFP* overexpression lines (micro-tom) were incubated with 10 μL of a *B. cinerea* suspension (5 × 10^5^ spores/mL) for 7 days prior to analysis. The disease symptoms were assessed and the lesion areas in the *SlBIR3-GFP* overexpression lines were approximately four-fold larger than in wild type (Figure 5A,B), indicating that *SlBIR3* suppresses resistance to *B. cinerea* infection in tomato. To verify this, detached leaves from a susceptible tomato cultivar, moneymaker, in which *SlBIR3* expression was silenced by VIGS, were incubated with 5 mm diameter mycelium plugs of *B. cinerea.* The silencing efficiency of *SlBIR3* gene is shown in Figure S6B and the disease symptoms were evaluated 4 days after the infection. As shown in Figure 5C,D, no significant difference was observed between *SlBIR3* silenced plant and the control on lesion areas in leaves. We also examined infections phenotypes in plants of the same genotype using a drop-inoculation method (Figure 5C,E), which confirmed that loss of *SlBIR3* function did not alter the disease resistance to *B. cinerea*.

**Figure 6:**
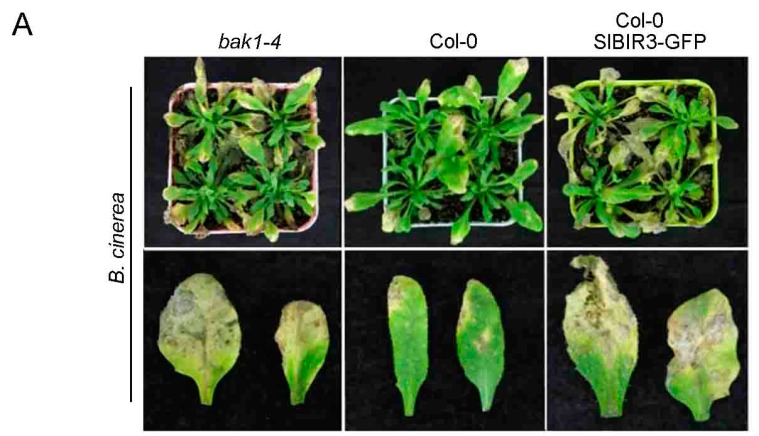

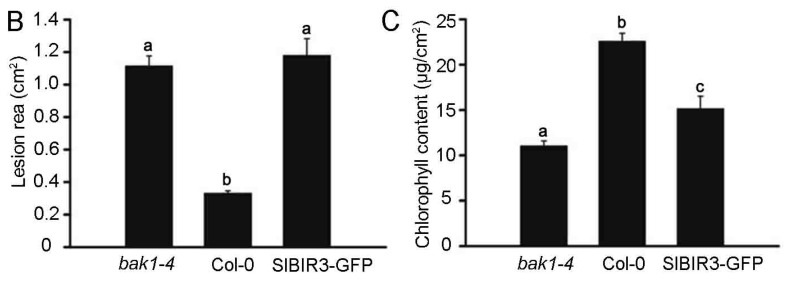
Heterologous expression of *SlBIR3* in *A. thaliana* suppresses resistance to *B.* *cinerea* (**A**) Disease phenotypes of *bak1-4*, Col-0 (wild type) and SlBIR3-overexpression plants (all Col-0 background) after spraying with a *B. cinerea* spore suspension, 7 days after the inoculation. The upper panel shows the whole plants and the lower panel shows detached leaves. (**B**) The lesion areas in plants shown in (**A**). The average lesion areas of 10 leaves were determined. (**C**) The total chlorophyll contents of leaves from the plants shown in (**A**). Chlorophyll was extracted from 10 rosette leaves infected by *B. cinerea* corresponding to leaves shown in (**A**). Data represent mean values ± SD. Significant differences are indicated by different letters according to a Student’s *t* test (*p* < 0.05). Experiments were performed three times with similar results.

To further confirm the role of *SlBIR3* in *B. cinerea* resistance, a *B. cinerea* spore suspension (5 × 10^5^ spores/mL) was sprayed on *A. thaliana* wild type, the *bak1-4*, mutant and *SlBIR3* overexpression lines (all Col-0 background) and the plants were placed in high humidity conditions under light for 7 days. We observed necrotic spots over the entire leaf surface of the *SlBIR3* overexpression lines and the *bak1-4* mutant, while the necrotic symptoms in the wild type leaves were restricted to the infection sites (Figure 6A). The lesion areas in the *SlBIR3* overexpression lines were also much larger than in wild type (Figure 6B), which correlated with the reduced chlorophyll levels caused by the *B. cinerea* infection (Figure 6C). These results suggest that *SlBIR3* suppresses resistance to *B. cinerea* infection in *A. thaliana*, which is consistent with its action in tomato. Taken together, these results suggest that overexpression of *SlBIR3* weakens the basal defense and impairs resistance to *B. cinerea* in tomato and *A. thaliana.*

### 2.6. SlBIR3 and SlSERK3 Control Cell Death in Tomato

**Figure 7:**
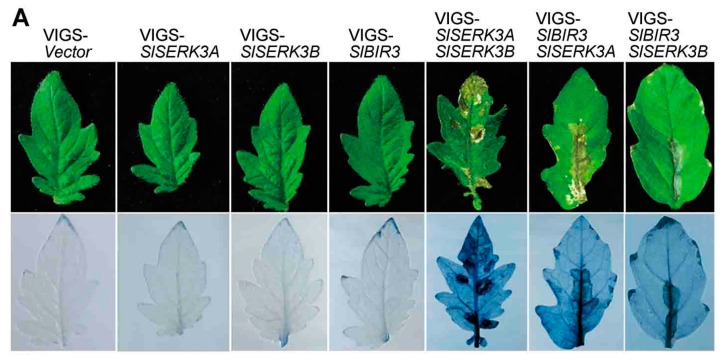

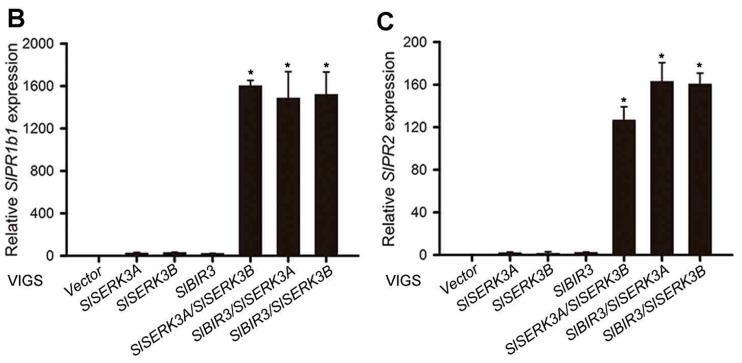
Co-silencing of *SlBIR3* and *SlSERK3* triggers cell death. (**A**) Phenotypes of individually silenced *SlSERK3A*, *SlSERK3B* and *SlBIR3* plant leaves and co-silenced *SlSERK3A*/*SlSERK3B*, *SlBIR3*/*SlSERK3A* and *SlBIR3*/*SlSERK3B* plant leaves (upper panel). The lower panel shows trypan blue staining of leaves corresponding to the upper panel. (**B**,**C**) The relative transcript levels of pathogenesis related genes, *SlPR1b1* and *SlPR2*, in leaves showed in (**A**), measured by real-time qRT-PCR, with *SlUBI3* used as the reference gene. The asterisks indicate significant differences compared to the control (VIGS-Vector) according to a Student’s *t* test (* *p* < 0.05). Experiments were performed three times with similar results.

It has been reported that co-silencing *SlSERK3A* and *SlSERK3B* in tomato leaves using VIGS triggers cell death [47]. To investigate whether SlBIR3 is involved in controlling cell death, we used VIGS to co-silence *SlSERK3* and *SlBIR3* in the tomato cultivar, Moneymaker. The silencing efficiency of the target genes (Figure S7A) was determined using real-time qRT-PCR. As shown in Figure 7A and Figure S7B, the cell death phenotype was observed in both *SlSERK3A/SlBIR3* and *SlSERK3B/SlBIR3* co-silenced plants, which is similar to that in *SlSERK3A*/*SlSERK3B* co-silenced plants. We observed no cell death symptoms in plants in which *SlSERK3A*, *SlSERK3B* or *SlBIR3* were silenced separately (Figure 7A and Figure S7B). Cell death was visualized by trypan blue staining (Figure 7A).

Another study showed that defense related genes are upregulated in *SlSERK3A*/*SlSERK3B* co-silenced plants [47] and so we measured the relative transcript levels of two pathogenesis genes, *SlPRb1* and *SlPR2*, in the leaves shown in Figure S7B, using real-time qRT-PCR. The expression levels of these two genes was significantly higher in both *SlSERK3A/SlBIR3B* and *SlSERK3B/SlBIR3* co-silenced leaves, and was similar to that in *SlSERK3A*/*SlSERK3B* co-silenced leaves (Figure 7B,C), which is consistent with the cell death results (Figure 7A). These results indicated that *SlBIR3* suppresses cell death together with *SlSERK3A* and *SlSERK3B* in tomato.

## 3. Discussion

Plant LRR-RLK proteins play key roles in the sensing of external biotic and abiotic stimuli, and in their conversion into internal chemical signals that regulate responses to ever changing environmental conditions [48]. According to their activation or catalytic mode of action, LRR-RLK proteins can be grouped into RD kinases and non-RD kinases [15], and the latter have usually been associated with plant innate immunity [15]. Previously, a small subfamily of BIR proteins were identified in *A. thaliana* and categorized as non-RD kinases [38]. Here, a tomato non-RD kinase gene, named *SlBIR3* (*Solyc*05g006570.1.1), an ortholog of *AtBIR3*, was shown to play roles in regulating PTI responses and controlling cell death.

A phylogenetic analysis revealed the widespread presence of the BIR proteins family in plants (Figure S2). Genomic DNA and complementary DNA sequences analyses showed that *SlBIR3* has only one exon, which is consistent with *AtBIR3* [38] and the closest orthologs from other plant species, indicating conserved exon composition (Figure S2). A domain analysis indicated that SlBIR3 has five leucine-rich repeats (LRR) regions, a transmembrane domain and a Ser/Thr cytoplasmic domain (Figure 1). This is also consistent with the domain structures of AtBIR3 and AtBIR2 [38] and is a typical and conserved signature motif of an LRR-RLK [48]. Previous studies demonstrated that LRR-RLK proteins lacking the conserved catalytic subdomain in the cytoplasmic domain usually do not have autophosphorylation and transphosphorylation activity in vitro [11,15,39,49,50]. Our results (Figure 1C and Figure 2) support the idea that SlBIR3 does not have autophosphorylation and transphosphorylation activity due to the absence of the RD motif in the subunit VIb domain. Previous studies showed that non-RD kinases can also be phosphorylated in vivo [50,51] and some of phosphorylation sites are critical for maintaining their functions [51,52]. In the current study, the phosphorylation of threonine residues in SlBIR3 was detected in vivo (Figure 3C) by immunoblotting, using a phosphothreonine antibody, which is consistent with previous studies of BIR2 [50].

It has been reported that functional BAK1 in *A. thaliana* and tomato, and members of the BIR proteins family in *A. thaliana* are mainly localized in the plasma membrane [16,38,39]. Domain analysis suggested that there is a signal peptide in the *N*-terminus of SlBIR3 (Figure 1A,B) and subcellular localization analysis indicated that SlBIR3 is mainly localized on the cell surface (Figure 2A,B). Taken together, these results suggest that SlBIR3 may interacts with BAK1 in vivo. Indeed, yeast two hybrid, co-IP and MBP pull down assays all confirmed that SlBIR3 can interact with SlBAK1 and AtBAK1 (Figure 3), which is consistent with previous reports that BIR proteins can interact with BAK1 in *A. thaliana* [38,41]. We noticed that SlBIR3 phosphorylation was greatly weakened in the absence of BAK1 (Figure 3C); however, it was detectable (Figure 3C), indicating that SlBIR3 can be targeted *in planta* by protein kinases other than BAK1. This conclusion is supported by previous studies in which BIR3 was identified in SERK1 immunoprecipitates using tandem mass-spectrometry [53].

Functional analysis of SlBIR3 overexpression or knockdown tomato and *A. thaliana* plants revealed that SlBIR3 participates in BAK1-regulated PTI and cell death pathways, but has weak effect on BR signaling. We found that overexpressing *SlBIR3* in tomato (micro-tom background) caused no significant phenotypic changes or to the expression levels of the BR responsive genes, *SlCPD* [47] and *SlDWARF* [54] (Figure S3A–D). This is consistent with a previous study of BIR2 in *A. thaliana* [38], and indicates that SlBIR3 has weak effect on plant growth and development through BR signaling. Micro-tom is a weak BR biosynthetic mutant and contains a mutation in the *D* gene (*CYP85A1*) [55]. Two orthologous genes in *A. thaliana* are *brassinosteroid-6-oxidase 1* (*BR6OX1*) and *brassinosteroid-6-oxidase 2* (*BROX2*) [56] and a BR-deficient *br6ox1 br6ox2* double mutant was used to study the BR biosynthesis mechanism [57]. The growth impairment of another weak BR-deficient *A. thaliana* mutant, *de-etiolated 2* (*det2*) [58,59], could be partially rescued by loss of *suppressor of BRI1* (*SBI1*) [59], *BIK1* [60], *BKI1* [61], and *protein phosphatase 2A* (*PP2A*) [62]; all components of the BR signaling pathway. Together, these studies suggest that BR-deficient micro-tom can provide a valuable resource for BR signaling research.

It was reported that overexpression of *SlBRI1* and *GmBRI1*, respectively, could partially complement the phenotype of the weak BR-insensitive mutant *bri1-5* [63,64,65] and overexpression of *SlSERK3* in the *BAK1* loss-of-function *A. thaliana* mutant *bak1-4* restored its sensitivity to exogenous BR [47]. We demonstrated that overexpressing *SlBIR3* in *A. thaliana* Col-0 did not result in any significant phenotypic changes (Figure S4) and that the BR sensitivity of *SlBIR3* transgenic lines was only slightly altered (Figure S5). These results suggest that SlBIR3 has no apparent but weak effect on BR signaling, although it has yet to be determined whether the other SlBIR proteins have functions in BR signaling.

In a microbe rich environment, plants mainly depend on innate immunity to defend themselves against invasion [66,67]. In our study, we observed that PTI signaling induced by flg22 was reduced in *SlBIR3* overexpression lines (Figure 4), indicating that SlBIR3 negative regulates PTI signaling. This is contrary to the function of its partner BAK1 [27] and that of the flagellin receptor FLS2 [68], but is consistent with its paralog, BIR2. This suggested that SlBIR3 may be a negative regulator of BAK1 in PAMP responses, but the response of the *SlBIR3* transgenic and knockdown lines to *Pst*DC3000 bacteria was unaffected (Figure S6), which is consistent with the phenotypes of knocking out AtBAK1 in *A. thaliana* [46] and knocking down SlBAK1 in tomato [47]. We propose that this may be due to the involvement of *SlBIR3* in cell death (Figure 5, Figure 6 and Figure 7).

The necrotrophic fungus, *B. cinerea*, a pathogen worldwide, has a wide range of hosts and causes severe damage to crops, especially tomato [69]. Previous studies reported that loss of function of *BAK1* [46] and *BIR2* [38] in *A. thaliana*, respectively, resulted in decreased resistance to *B. cinerea*. In our study, we found that role of SlBIR3 in resistance to *B. cinerea* is opposite to that of BIR2 [38], indicating functional differentiation of BIR during evolution. This result also supports the idea that *SlBIR3* is a negative regulator of BAK1 in PAMP responses. 

Several studies reported that BAK1 plays important roles in spontaneous cell death. Loss-of-function of BAK1 and BAK1-like 1 (BKK1) together causes a spontaneous cell death phenotype in a salicylic acid (SA) dependent manner, which could be rescued by loss-of-function of suppressor of BAK1 BKK1 (SBB1), and partial loss of endoplasmic reticulum quality control (ERQC) components, pep receptor 1 (PERP1) and PEP RECEPTOR 2 (PEPR2) [35,36,70]. Loss-of-function of BAK1 and BIK1 together was also reported to induce spontaneous cell death, which was rescued by loss-of-function of phytoalexin deficient 4 (PAD4) and salicylic acid induction deficient 2 (SID2), respectively [71]. The role of BAK1 in controlling spontaneous cell death, together with other RLKs, was further verified by our results showing that co-silencing of *SlBIR3*/*SlSERK3A* and *SlBIR3*/*SlSERK3B* lead to leaf necrosis, which was similar to the co-silencing of *SlSERK3A*/*SlSERK3B* by VIGS in tomato [47]. This suggests that *SlBIR3* negatively regulates spontaneous cell death and that *SlSERK3s* is critical for the development of *SlBIR3* knockdown mutant (Figure 7 and Figure S7). BIR1 regulates temperature-dependent spontaneous cell death with BON1 [40] and the spontaneous cell death phenotype in loss of BIR1 function mutant, *bir1*, could be rescued by loss of function of BAK1 [41]. Cell death in the *bir2* mutant was only observed after fungal infection [38]. We conclude that the cell death pathway controlled by SlBIR3 is distinct from that regulated by BIR1 and BIR2. It will be interesting to test whether the components that can rescue BAK1-related spontaneous cell death is involved in SlBIR3-controlled cell death and whether SlBIR3 is involved in BAK1-regulated other signaling, such as that associated with abscisic acid (ABA) signaling in guard cells [72].

## 4. Materials and Methods

### 4.1. Plant Materials and Growth Conditions

Plants used in this study were wild-type *Arabidopsis thaliana* (ecotype Col-0); the *A. thaliana* mutant, *bak1-4* (SALK_116202); tobacco (*Nicotiana benthamiana*); tomato (*Solanum lycopersicum*, cvs. moneymaker and micro-tom). The mutant was obtained from the Arabidopsis Biological Resource Center (ABRC, http://www.arabidopsis.org/). For tomato transformation we used cotyledons [73] and for *A. thaliana* transformation we used a *Agrobacterium tumefaciens*-mediated floral dip protocol [74]. The *A. thaliana* seeds were sterilized with 15% (*v*/*v*) NaClO containing 0.1% Triton X-100 (Sigma-Aldrich, Saint Louis, MO, USA) for 15 min and washed four times with sterile water. 33 mg/L Hygromycin (Roche, Mannheim, Germany) was used to screen T1 SlBIR3 transgenic plants. Plants were grown in a greenhouse at 21–23 °C, a relative humidity of 65%, and 16 h light (150–200 μmol·m^−2^·S^−2^)/8 h dark on soil or 1/2 Murashige and Skoog (MS) medium [36] (1 M NaOH was used to adjust pH to 5.8) containing 7% agar and 2% sucrose.

### 4.2. Tobacco Transient Transformation

The coding sequence of *SlBIR3* was subcloned into a CaM35SGFP vector [75] forming a construct encoding SlBIR3 with a C-terminal GFP, driven by the CaMV35S promoter. The primers used are listed in Table S1. An *A. tumefaciens* (GV3101) culture transformed [76] with the above vector was shaken at 28 °C, 200 rpm for overnight. The OD_600_ of the culture was adjusted to 0.2 with infiltration medium (27.8 mM d-glucose, 2.5 mM MES, pH 5.8 (adjusted by addition of 1 M NaOH), 0.1 mM Na_3_PO_4_·12H_2_O, 5 mM acetosyringone). The culture A was then left at room temperature for at least 3 h before infiltration into tobacco leaves [76]. The GFP signal in the transformed leaves was detected three days after infiltration using a fluorescence microscope (BX51, Olympus, Tokyo, Japan) under blue light. Images were processed by the ImageJ software.

### 4.3. Autophosphorylation and Transphosphorylation In Vitro

DNA sequences encoding the cytoplasmic domain of BAK1, mBAK1 (L317E), SERK5 and SlBIR3 were amplified by PCR and subcloned into the pFLAG-MAC vector (Sigma-Aldrich Saint Louis, MO, USA). The primers used are listed in Table S1. Each of the resulting constructs was transformed into *E. coli* BL21(DE3)pLysS (Transgene, Beijing, China). The resulting recombinant proteins were purified as previously described and the autophosphorylation and their transphosphorylation activities in vitro were determined as previously described [77], using anti-FLAG (1:5000) (Transgene, Beijing, China, Cat. #HT201-01) and anti-pThr (1:2000) (CST, Danvers, MA, USA, Cat. #93815) antisera.

### 4.4. Yeast Two-Hybrid Assay

To verify interactions between SlBIR3 and BAK1, the pGADT7 plasmid (Clontech, Mountain View, CA, USA) containing the cytoplasmic domain of SlBIR3, and pGBKT7 plasmid (Clontech, Mountain View, CA, USA) containing the cytoplasmic domain of AtBAK1 or SlBAK1 were co-transformed into yeast (*Saccharomyces cerevisiae*) strain AH109 (Clontech, Mountain View, CA, USA). The resulting transformants were grown on SD/-Leu-Trp (DDO) medium at 30 °C for 3 days. Individual colonies were picked from the plates and subcultured in 2 mL of SD/-Leu-Trp liquid medium at 30 °C for 24 h. The cultured cells were spotted on SD/-Leu-Trp, SD/-Leu-Trp-His-Ade (QDO, and SD/-Leu-Trp-His-Ade + X-α-Gal (QDO + X-α-Gal) medium and incubated at 30 °C. Plates were photographed after 4 days of incubation.

### 4.5. Protein Extraction, Co-IP and Western Blot Analyses

Ten micrograms of total protein extracted from the transgenic plants or the infected tobacco leaves of the same age were loaded on SDS-PAGE gels for the western blot analyses with anti-GFP (1:5000) antibody (Transgene, Beijing, China, Cat. #HT801-01) as previously described [78]. *A. thaliana* seedlings used for co-IP were grown in shaking liquid culture [9] without 1 mM 24-epiBL (Sigma-Aldrich, Saint Louis, MO, USA) treatment. Total protein isolation, immunoprecipitation, and immunoblot analysis were performed as previously described in [78] with anti-GFP (1:5000) (Transgene, Beijing, China, Cat. #HT801-01), anti-BAK1 (1:5000) (Genscript, Wuhan, China, Order ID. #7178897-1) and anti-pThr (1:2000) (CST, Danvers, MA, USA, Cat. #93815) antibodies.

### 4.6. In Vitro Pull-Down Assay

The cytoplasmic domains of *SlBAK1* and *AtBAK1* were amplified and subcloned into the pFLAG-MAC vector (Sigma-Aldrich, Saint Louis, MO, USA). The cytoplasmic domains of *SlBIR3* and *PUB13* were amplified and subcloned into the pMal-C2 vectors (New England Biolab, Ipswich, MA, USA), to generate MBP tagged proteins. The primers used are listed in Table S1. Each of the constructs was transformed into *E. coli* BL21(DE3)pLysS. Recombinant SlBAK1-FLAG and AtBAK1-FLAG were purified with Anti-FLAG M2 Affinity Gel beads (Sigma-Aldrich, Saint Louis, MO, USA) according to manufacturer’s protocols, and MBP, MBP-SlBIR3, MBP-PUB13 fusion proteins were purified after immobilization with amylose resin (New England Biolab, Ipswich, MA, USA, Cat. #DP301-01) following standard protocols. Three micrograms of FLAG fusion proteins were preincubated with 10 μL prewashed amylose resin in 120 μL incubation buffer (1 mM NaCl, 20 mM MgCl_2_, 0.5% Triton X-100, and 0.1 M HEPES, pH 7.2 (adjusted by addition of 1 M NaOH) for 1 h at 4 °C. After centrifugation at 5000× *g* for 5 min at room temperature the resin was collected and washed 5 times with incubation buffer. The pull-down proteins were detected by Western blot analysis with an anti-FLAG antibody (1:5000) (Transgene, Beijing, China, Cat. #HT201-01).

### 4.7. Root and Hypocotyl Growth Analyses

After surface sterilization with 15% NaClO containing 0.1% Triton X-100 (Sigma-Aldrich, Saint Louis, MO, USA), seeds were stored at 4 °C for two days and then planted on 1/2 MS medium supplemented with 0.7% (*w*/*v*) agar, 1% (*w*/*v*) sucrose and different concentrations of 24-epiBL. The plates were then placed vertically at 22 °C and the root length was measured seven days after germination under long-light conditions (16 h/8 h), and the hypocotyl length was measured five days after germination in the dark [33].

### 4.8. Seedling Growth Inhibition Assay

The seedling growth inhibition assays of the mentioned seedlings were performed as previously described [79].

### 4.9. VIGS of SlBIR3 in Tomato

A 400 bp of *SlBIR3* corresponding to 1–400 bp of *SlBIR3* was amplified from cDNA derived from tomato (cv. moneymaker) and subcloned into *Eco*RI-*Bam*HI-digested pTRV2 vector [45], generating the construct pTRV2-*SlBIIR3*, which was used to silence *SlBIR3* expression in tomato. A 409 bp of *SlPDS*, corresponding to 858–1266 bp of *SlPDS*, was also amplified from the tomato (cv. moneymaker) cDNA and subcloned into *Eco*RI-*Bam*HI-digested pTRV2 vector forming the construct pTRV2-*SlPDS*, which was used to silence *SlPDS* and as a positive control. The empty pTRV2 vector was used as a negative control. VIGS constructs to silence *SlSERK3A* and *SlSERK3B* separately, and for both genes *SlSERK3A*/*SlSERK3B* together, as well as fragments of *SlSERK3A* (152 bp), *SlSERK3B* (174 bp) and *SlSERK3A/B* (177 bp), were amplified from tomato (cv. Moneymaker) cDNA and subcloned into *Bam*HI-*XhoI*-digested pTRV2 vector, forming the constructs pTRV2-*SlSERK3A*, pTRV2-*SlSERK3B* and pTRV2-*SlSERK3A/B* [47]. VIGS constructs for both genes *SlBIR3*/*SlSERK3A* and *SlBIR3*/*SlSERK3A*, fragments of *SlBIR3* (400 bp), as mentioned above, were subcloned into *Eco*RI-*BamHI*-digested pTRV2-*SlSERK3A* and pTRV2-*SlSERK3B* vectors, respectively, forming the constructs pTRV2-*SlBIR3*/*SlSERK3A* and pTRV2-*SlBIR3*/*SlSERK3B*, respectively [35]. The recombinant constructs and the empty vector were each transformed into *A. tumefaciens* strain GV3101. The primers used to generate these constructs are listed in Table S1. The tomato and the leaf infiltration method used for the VIGS assay was as previously described [45].

### 4.10. Pathogen Preparation and Inoculation

*Pseudomonas syringae* pv. *tomato* DC3000 (*Pst*DC3000) infections were performed as previously described [80]. *Botrytis cinerea* strain ACCC30091 was obtained from the Agricultural Culture Collection of China (http://www.accc.org.cn/htdocs/epages.asp?id=13) and the strain was rejuvenated in tomato fruit, separated and grown in potato dextrose agar (PDA) medium [81]. *B. cinerea* spores were washed down from the PDA plate with distilled water. After gentle shaking (100 rpm) at 26 °C for 30 min, the suspension was filtrated with absorbent cotton gauze and then centrifuged at 800× *g* for 3 min at room temperature. The supernatant was poured off and the collected spores were gently resuspended in distilled water containing 0.025% Tween and 2% glucose. The spores were counted using a hemacytometer under a light microscrope and the spore suspension (5 × 10^5^ spores/mL) were prepared. For *B. cinerea* infections of tomato, each detached leaf from 4-week-old tomato was incubated with 10 µL *B. cinerea* suspension using a drop-inoculation method or 5 mm *B. cinerea* diameter mycelium plugs [69,81]. The detached leaves were placed in a chamber at 21–23 °C, 16 h light/8 h dark for 7 days in the case of drops incubation and for 4 days in the case of mycelium plug incubation. For *B. cinerea* infections of *A. thaliana*, spore suspension (5 × 10^5^ spores/mL) containing 0.025% Tween and 2% glucose were sprayed on 5-week-old plants, and the plants were then grown in 100% relative humidity and at 24 °C under light for 7 days. Leaf areas were calculated using ImageJ software.

### 4.11. Semi-Quantitative and Real-Time Quantitative RT-PCR Analyses

To measure the expression levels of *SlBIR3*, *SlDWARF* and *SlCPD* in the transgenic tomato lines, total RNA was extracted from five-week-old plants. To measure the expression levels of *DWF4* and *CPD* in *A. thaliana* transgenic lines, total RNA was extracted from 14-day-old seedlings grown on 1/2 MS medium supplemented with 0.7% (*w*/*v*) agar and 2% (*w*/*v*) sucrose. For the flag22 sensitivity test, total RNA was extracted from 14-day-old seedlings grown on 1/2 MS medium supplemented with 1% (*w*/*v*) sucrose with or without a 3 h 100 nM flg22 (Genscript, Wuhan, China) treatment. For VIGS assay, the inoculated leaves were used for extracting total RNA. In each case, total RNA was extracted with RNAiso Plus kit (TaKaRa, Dalian, China) according to the manufacturer’s protocol and transcribed to cDNA with Transcriptor First Strand cDNA Synthesis Kit (Roche, Mannheim, Germany) according to the manufacturer’s protocol after treatment with DNase I (Thermo Scientific, Waltham, MA, USA). For semi-quantitative RT-PCR, PCR was performed using a 2× Es Taq MasterMix (Cwbio, Beijing, China). For real-time quantitative RT-PCR, PCR was performed using a SYBR Green Master Mix kit (Vazyme, Nanjing, China) with the above cDNA, and the relative quantitative expression of *FRK1*, *SlPR1b1*, *SlPR2.0*, *SlSERK3A*, *SlSERK3B*, and *SlBIR3* were detected using ABI StepOne plus (ABI, Foster, CA, USA) with *ACTIN2* or *SlUBI3* as a normalized control and calculated using the delta-delta Ct method [82]. The PCR reaction was performed in a 20-μL volume in 8-well tubes heated for 1 cycle of 5 min at 95 °C, 40 cycles of 10 s at 95 °C and 30 s at 60 °C, and final a dissociation stage. For each primer combination, the specificity and the amplification efficiency was checked by melting-curve analysis and standard-curve analysis, respectively. The primers used are listed in Table S1.

### 4.12. Trypan-Blue Staining and Chlorophyll Measurement

Trypan-blue Staining used for detecting cell death was performed as previously described [83]. Chlorophyll content measurements were performed as previously described [84] with modifications. Briefly, 10 rosette leaves infected by *B. cinerea* were placed in a vial containing 15 mL 80% acetone and chlorophyll was extracted into the fluid without grinding at room temperature by incubating for 24 h under dark condition. The supernatant from the vial was made up to 20 mL with 80% acetone. The absorbance of the mixture was measured using ultraviolet spectrophotometer (UV-1750, Shimadzu, Tokyo, Japan) and the total chlorophyll (chlorophyll a + b) was calculated as described in [84].

### 4.13. Sequence Analyses

The protein sequences were aligned using the softwares ClustalX 2.0 and Bioedit 7.0.9.0. The protein sequences used for the phylogenetic analysis were used to search the plant membrane protein database (http://aramemnon.uni-koeln.de/) and aligned using ClustalW2.0 (http://www.ebi.ac.uk/Tools/msa/clustalw2/). The phylogenetic tree was generated by PhyML, then viewed and edited using TreeDyn (http://www.phylogeny.fr/advanced.cgi).

## 5. Conclusions

*Solyc*05g006570.1.1 encodes SlBIR3, which is the *A. thaliana* ortholog of AtBIR3. Amino acid sequence and kinase activity analyses indicated that SlBIR3 is a membrane localized non-RD kinase, which interacts with, and could be phosphorylated by, BAK1. Overexpression of *SlBIR3* in plants suppresses flg22 induced PAMP responses, enhances the susceptibility to *B. cinerea* infection, but has weak effect on BR signaling. The use of VIGS with TRV-RNA2 containing fragments from *SlBIR3* and *SlSERK3* revealed that *SlBIR3* suppresses cell death in tomato together with *SlSERK3s*.

## Supplementary Materials

Supplementary materials can be found at www.mdpi.com/1422-0067/18/9/1966/s1.
