# Supplementary material for: SlBIR3 Negatively Regulates PAMP Responses and Cell Death in Tomato
Source: Int J Mol Sci. 2017 Sep 13;18(9):1966. doi: 10.3390/ijms18091966 (PMC5618615; doi:10.3390/ijms18091966)
Supplement: Supplementary file 1 [file ijms-18-01966-s001.zip › ijms-214996 supplement/Supplemental Figures S1-S7.docx]

## Supplemental Figures


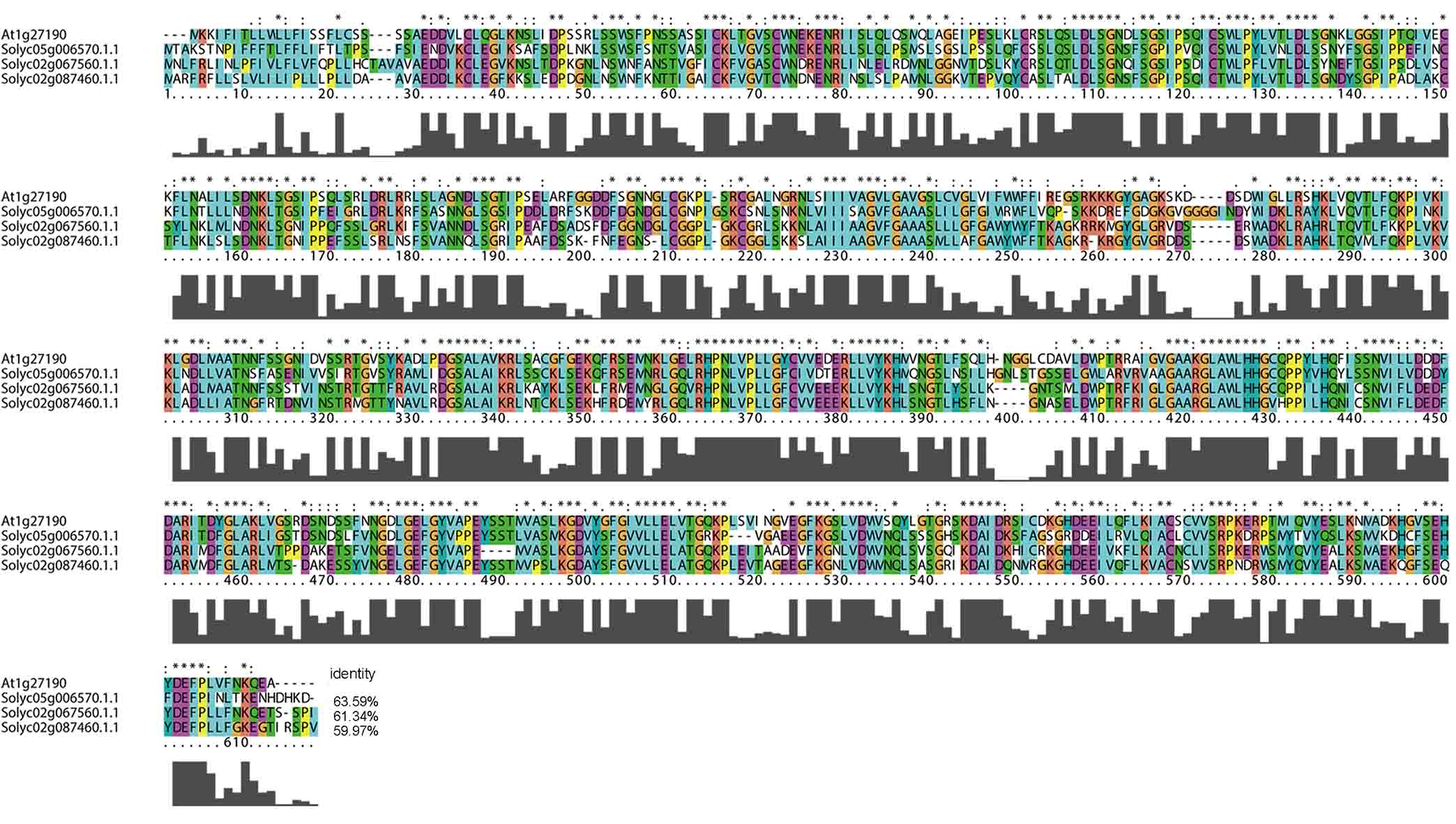


**Figure S1.** Protein sequence alignment of *Arabidopsis* BIR3 (At1g27190), tomato *Solyc*05g006570.1.1, *Solyc*02g067560.1.1 and *Solyc*02g087460.1.1.


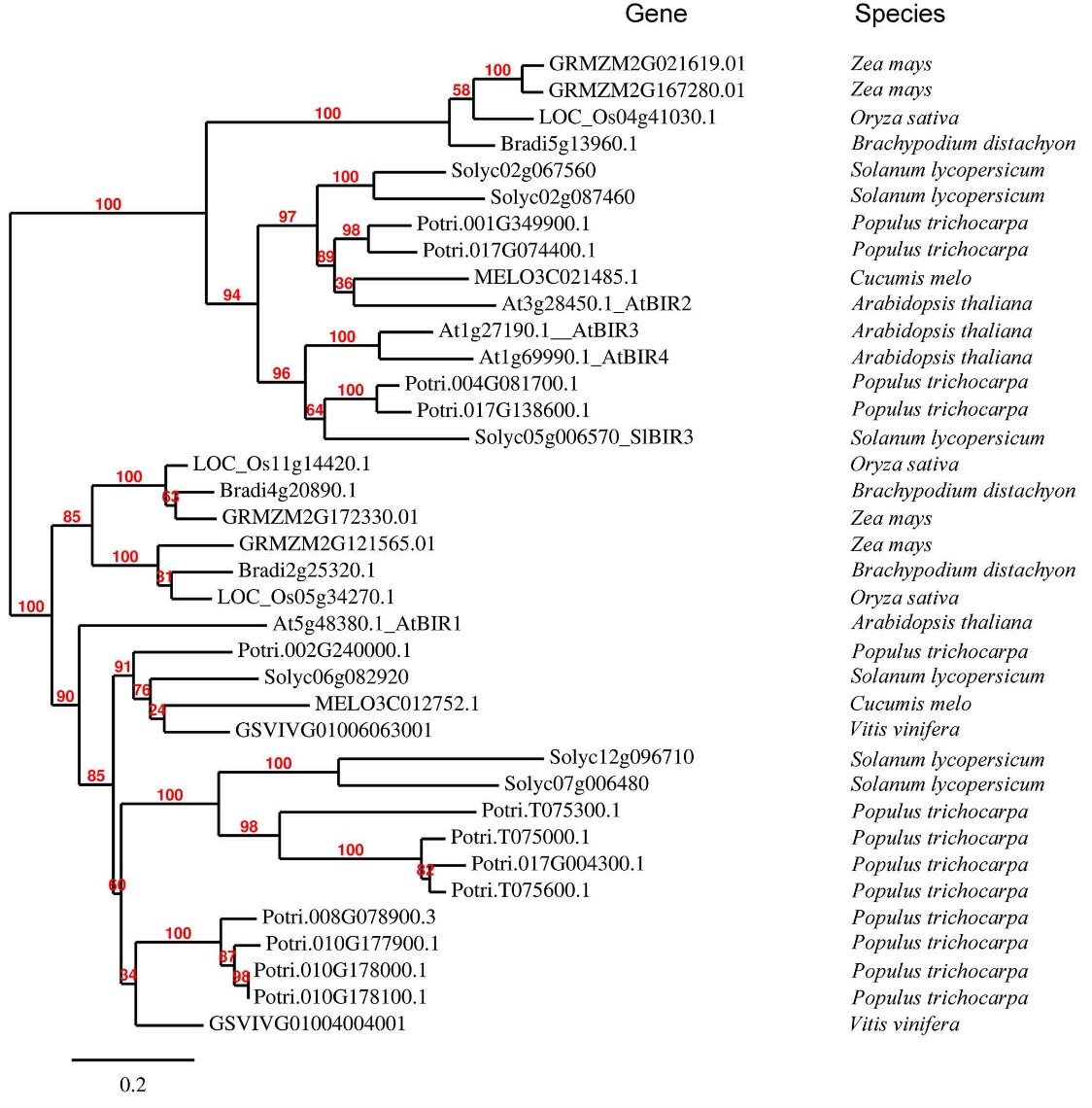


**Figure S2.** Phylogenetic analysis of members of the BIR proteins family from *S. lycopersicum*, *A. thaliana* and other plant species.


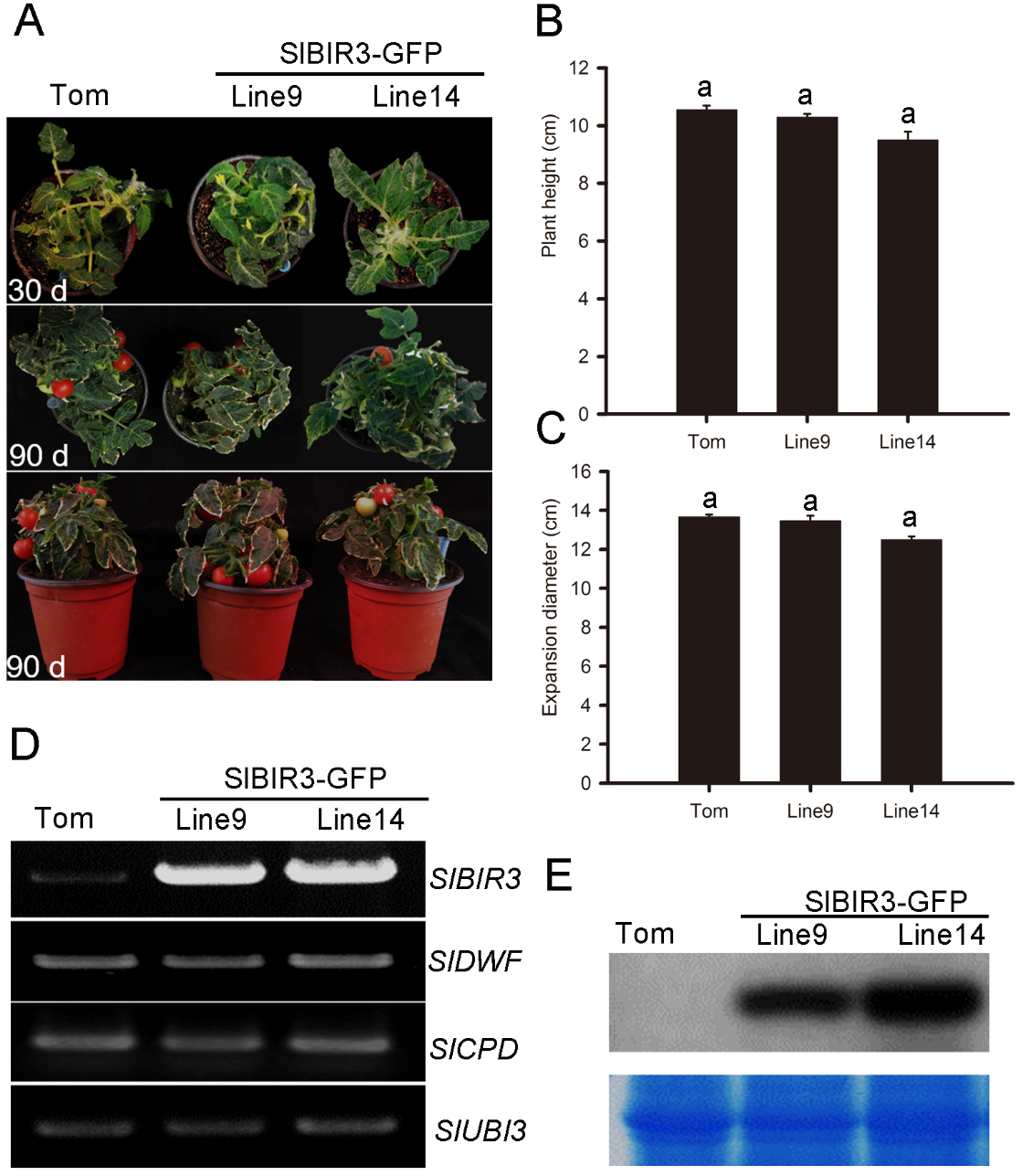


**Figure S3.** Overexpressing *SlBIR3* has weak effect on the growth and development and BR signaling in tomato cv. Micro-Tom. (A) Phenotypic characteristics of SlBIR3-GFP transgenic tomato lines 9 and 14. The photograph shows 30-day-old plants in the upper panel. The middle and lower panels show the same plants at 90 days. **(B-C)** Quantitative analysis of plant height and expansion diameter of the SlBIR3 transgenic lines 9 and 14. The average plant height and expansion diameter of six 90-day-old plants were measured. Data are the mean values ± SD. No significant differences between the lines was observed, as indicated by the same letter according to one-way ANOVA analysis and a Student’s *t* test (*p*<0.05). **(D)** Semi RT-PCR analysis showing the relative expression level of *SlBIR3*, as well as the BR signaling marker genes *SlCPD* and *SlDWARF*. *SlUBI3* (GenBank reference number, NM_001346406) was used as the reference gene. **(E)** Western blot analysis showed the expression level of the recombinant protein SlBIR3-GFP, detected with an anti GFP antibody. CBB staining shows the loading control.


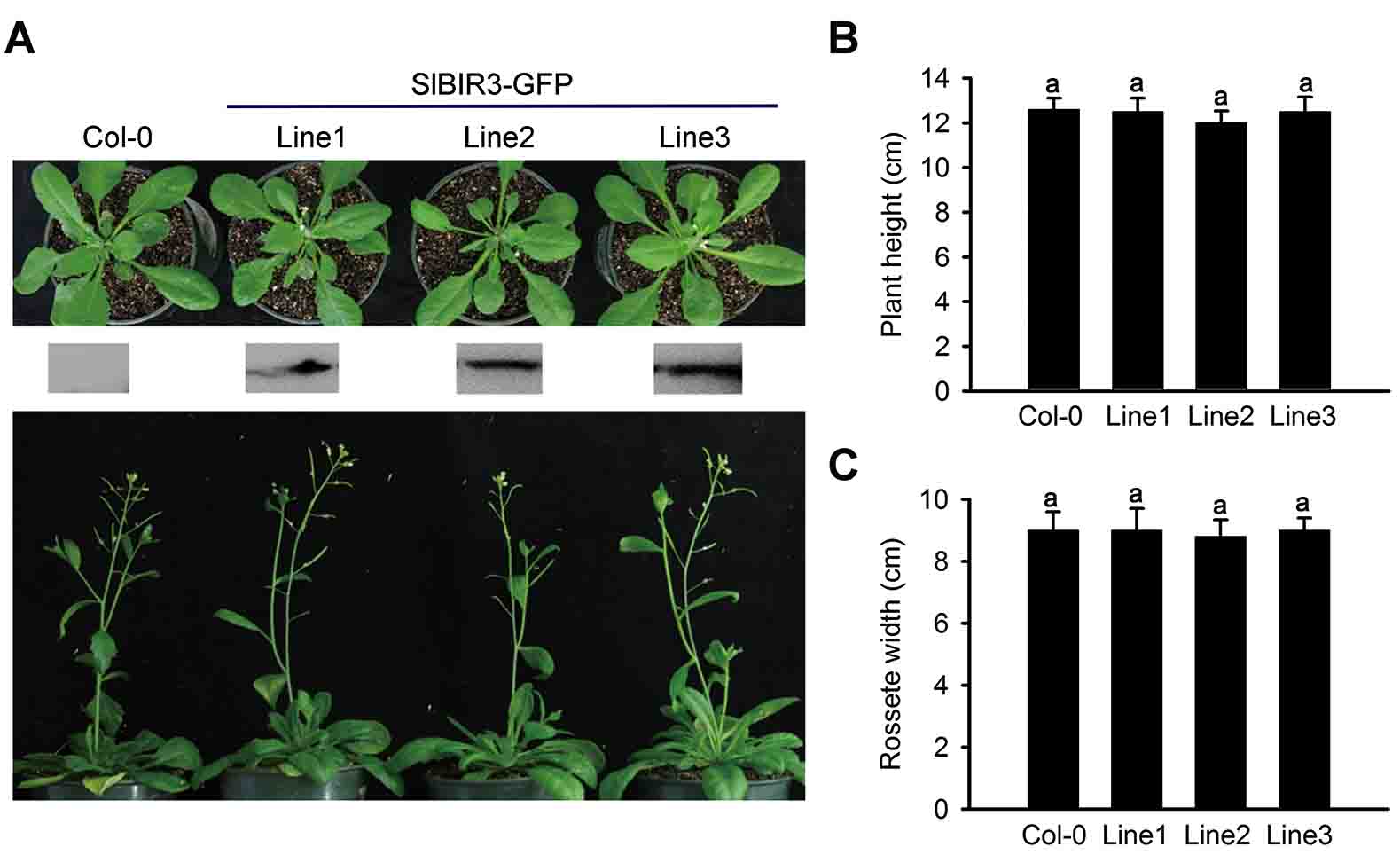


**Figure S4.** Overexpressing *SlBIR3* in *A. thaliana* has no significant effect on the growth and development. (A) Phenotypic characteristics of SlBIR3-GFP transgenic lines 1, 2 and 3 in the Col-0 background. The upper panel shows three-week-old plants. The Western blot images in the middle panel show the level of recombinant protein. The lower panel shows the same plants as those in the upper panel after four weeks. (B-C) Quantitative analysis of plant height and rosette width of the SlBIR3 transgenic plant lines 1, 2 and 3. Average plant height and rosette width were measured of ten plants at five weeks after planting. Data are mean values ± SD. No significant differences between the lines was observed, as indicated by the same letter according to a Student’s *t* test (*p*<0.05).


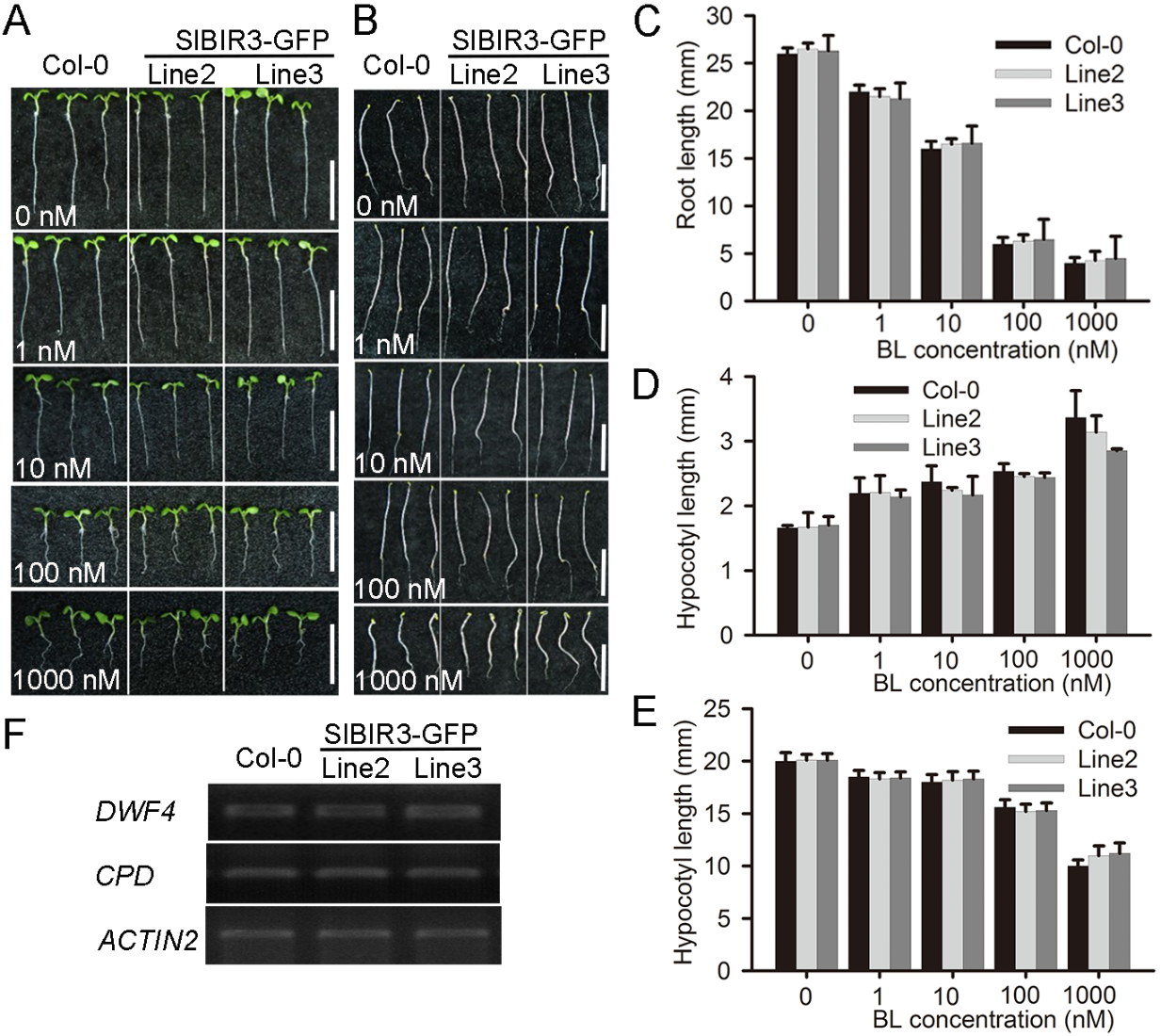


**Figure S5.** Overexpressing *SlBIR3* in *A. thaliana* has weak effect on BR signaling*.* Col-0 and SlBIR3-overexpression seedlings grown on 1/2 MS medium for seven days in the light (A) and five days in the dark (B) with different concentrations of 24-epiBL. Scale bars, 10 mm (C) Root length of Col-0 and SlBIR3-overexpression seedlings corresponding to (A). (D) Root length of Col-0 and SlBIR3-overexpression seedlings corresponding to (A). (E) Hypocotyl length of Col-0 and SlBIR3-overexpression seedlings corresponding to (B). At least 20 plants were measured. (F) The relative expression level of *CPD* or *DWF4* in Col-0 and SlBIR3-overexpression seedlings. Relative expression level of *CPD* and *DWF4* was measured by semi RT-PCR, with *ACTIN2* used as the reference gene. Data are mean values ± SD.


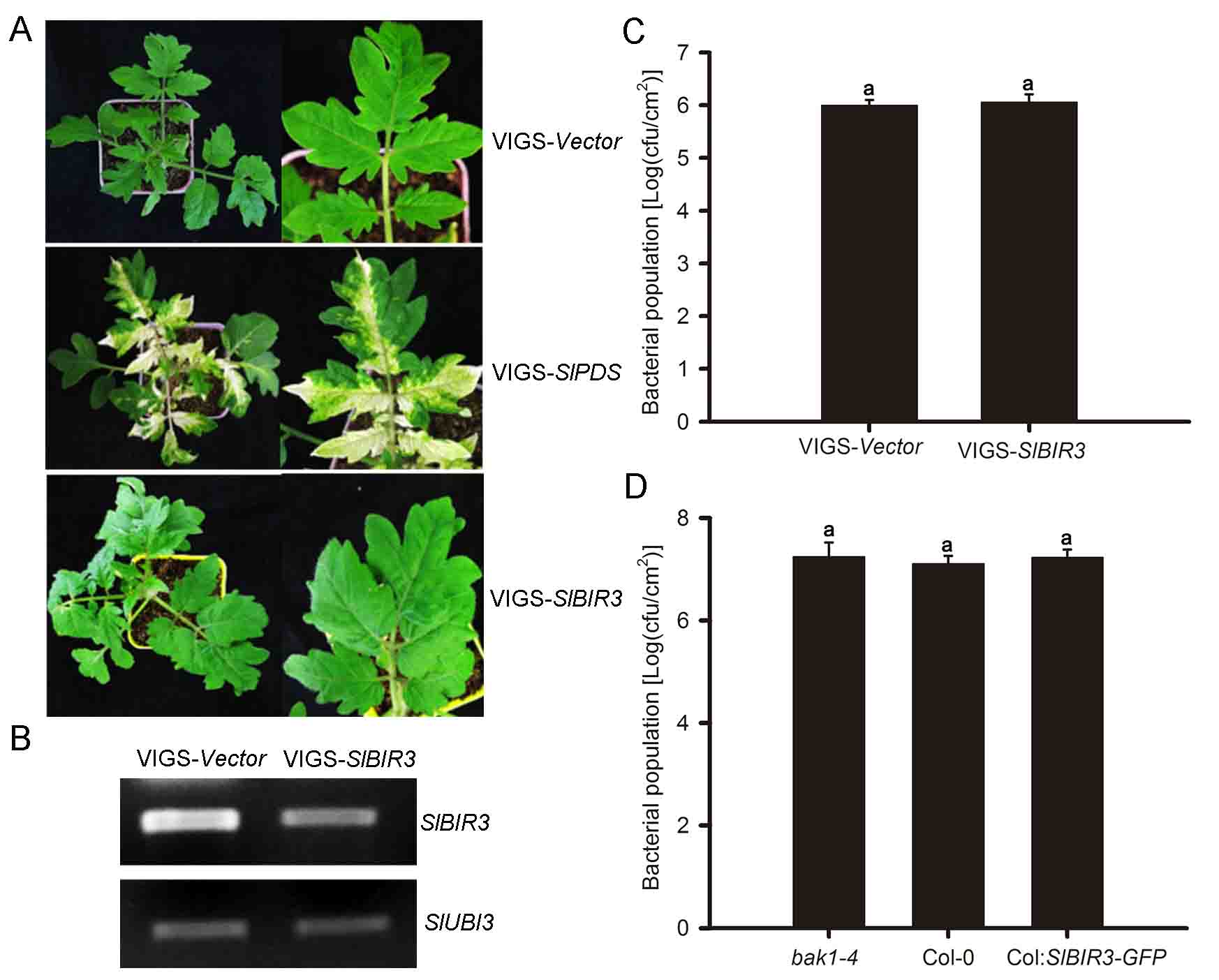


Figure S6. Both *SlBIR3* silenced tomato cv. Moneymaker plants and *SlBIR3* overexpressing *A. thaliana* are insensitive to *Pst*DC3000 treatment. (A) Phenotypic characteristics of plants infiltrated with *Agrobacterium* carrying different vectors. The photographs were taken four weeks after the infiltration. VIGS-*vector* and VIGS-*SlPDS* were used as negative and positive controls, respectively. VIGS-*SlBIR3* shows the *SlBIR3* silenced plants. (B) The silencing efficiency of *SlBIR3* in tomato cv. Moneymaker. The relative expression level of *SlBIR3* in leaves of the indicated plants corresponding to (A) was analyzed by semi RT-PCR with *SlUBI3* as the reference gene. (C) Quantitative analysis of the number of *Pst*DC3000 cells in *SlBIR3* silenced tomato cv. Moneymaker plants. Plant leaves indicated in (B) were infiltrated with a *Pst*DC3000 suspension. The bacterial growth in the leaves was analyzed after 4 days of incubation. Three independent experiments were performed. Data are the mean values ± SE. (D) Quantitative analysis of the number of *Pst*DC3000 cells in the indicated plant leaves. Five-week-old *bak1-4* mutant, Col-0 and SlBIR3-GFP transgenic plants (Col-0 background) were infiltrated with a *Pst*DC3000 suspension. 4 days post-infection, the bacterial growth in the indicated plants was measured. Three independent experiments were performed. Data are the mean values ± SE. No significant differences were observed between the lines as indicated by the same letter according to a Student’s *t* test (*p*<0.05).


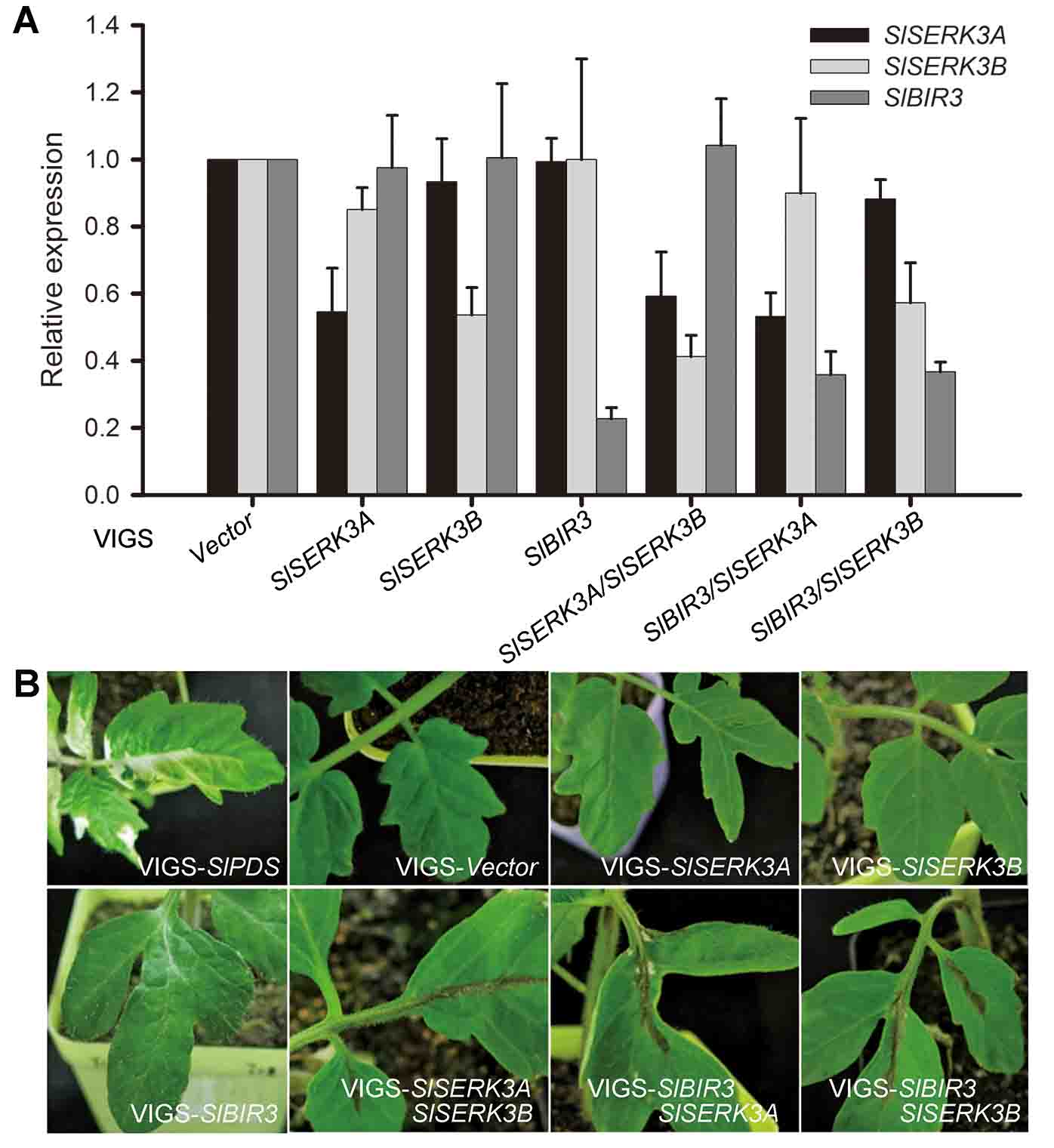


**Figure S7.** Co-silencing of *SlBIR3* and *SlSERK3* triggers cell death. (A) The target gene silencing efficiency in leaves shown in (B) was measured by real time quantitative RT-PCR with *SlUBI3* used as the reference gene. (B) Phenotypes of individually silenced *SlSERK3A*, *SlSERK3B* and *SlBIR3* plants and co-silenced *SlSERK3A*/*SlSERK3B*, *SlBIR3/SlSERK3A* and *SlBIR3/SlSERK3B* plants.
