# Supplementary material for: SlBIR3 Negatively Regulates PAMP Responses and Cell Death in Tomato
Source: Int J Mol Sci. 2017 Sep 13;18(9):1966. doi: 10.3390/ijms18091966 (PMC5618615; doi:10.3390/ijms18091966)
Supplement: Supplementary file 1 [file ijms-18-01966-s001.zip › ijms-214996 supplement/Supplemental Table S1.docx]

### Table S1. Primers used in this study

| SlBIR3-F+ BamHI | GAGCTCGGTACCCGGGGATCCATGACAGCAAAATCAACTAACCCC | for pCAM35S-GFP |
| --- | --- | --- |
| SlBIR3-R+SalI | CTTGCTCACCATGGTGTCGACATCTTTATGATCATGATTTTCCTTGG |  |
| (m)BAK1CD-F+HindIII | GACGATGACAAAGTCAAGCTTCGAAGGAAAAAGCCGCAG | for pFLAG-Mac |
| (m)BAK1CD-R+KpnI | CTATCTAGATCTGCAGGTACCTCTTGGACCCGAGGGGTAT |  |
| SERK5CD-F+HindIII | GACGATGACAAAGTCAAGCTTCTGAGAAGAAAACTGCAGGGTCAC |  |
| SERK5CD-R+KpnI | CTATCTAGATCTGCAGGTACCTCTTGGCCCCGAGGGGTA |  |
| SlBIR3CD-F+HindIII | GACGATGACAAAGTCAAGCTTAGATGGTTTTTAGTTCAGCCTAGTAAG |  |
| SlBIR3CD-R+EcoRI | TCTGCAGGTACCCGGGAATTCATCTTTATGATCATGATTTTCCTTGG |  |
| SlBAK1CD+F+ Hind III | GACGATGACAAAGTCAAGCTTCGTCGAAGGAAACCGGAAG |  |
| SlBAK1CD +R+ EcoRI | TCTGCAGGTACCCGGGAATTCTCTTGGCCCTGATAACTCATCG |  |
| BAK1CD-F+EcoRI | CCGGAATTCCGAAGGAAAAAGCCGCAG | for pGBKT7 |
| BAK1CD-R+BamHI | CGCGGATCCTCTTGGACCCGAGGGGTAT |  |
| SlBAK1CD-F+EcoRI | TATGGCCATGGAGGCCGAATTCGCGATTTTGCTTGCTTGGTG |  |
| SlBAK1CD-R+BamHI | GCCGCTGCAGGTCGACGGATCCTCTTGGCCCTGATAACTCATCG |  |
| SlBIR3CD-F+EcoRI | GGAGGCCAGTGAATTCAGATGGTTTTTAGTTCAGCCTAGTAAG | for pGADT7 |
| SlBIR3CD-R+BamHI | CGAGCTCGATGGATCCATCTTTATGATCATGATTTTCCTTGG |  |
| PUB13-F+EcoRI | CCGGAATTCATGGAGGAAGAGAAAGCTTCTG | for p Mal-c2 |
| PUB13-R+XbaI | ACGC GTCGACAGTATCTGCAGCTTCTGTGGTG |  |
| Pmal-SlBIR3CD+F+BamHI | AGGATTTCAGAATTCGGATCCAGATGGTTTTTAGTTCAGCCTAGTAAG |  |
| Pmal-SlBIR3CD+R+SalI | CCAAGCTTGCCTGCAGGTCGACATCTTTATGATCATGATTTTCCTTGG |  |
| 121-gfp-SlBIR3-F+XbaI | GAGAACACGGGGGACTCTAGAATGACAGCAAAATCAACTAACCCC | for pBI121-GFP |
| 121-gfp-SlBIR3-R+KpnI | GCCCTTGCTCACCATGGTACCATCTTTATGATCATGATTTTCCTTGG |  |
| SlBIR3-F+EcoRI | GTGAGTAAGGTTACCGAATTCATGACAGCAAAATCAACTAACCC | for VIGS |
| SlBIR3-F+BamHI | CGTGAGCTCGGTACCGGATCCTGCTGGAGAGATCGAGATTGAC |  |
| SlPDS-F+EcoRI | CGG TCT AGA GGC ACT CAA CTT TAT AAACC |  |
| SlPDS-R+ BamHI | CGG GGA TCC CTT CAG TTT TCT GTC AAA CC |  |
| SlSERK3A-F+BamHI | CGCGGATCCGGGCCAAGATGATCTTTCATT |  |
| SlSERK3A-R+XhoI | CCGCTCGAGCTGACACATATACCATTTAACCCC |  |
| SlSERK3B-F+BamHI | CGCGGATCCGGCCAAGATGATCTTTCAGC |  |
| SlSERK3B-R+XhoI | CCGCTCGAGATGTCATCAAAATTCCTGACATAC |  |
| SlSERK3AB-F+BamHI | CGCGGATCCAGGAGTATGAAGCAGTTGTTGGG |  |
| SlSERK3AB-R+XhoI | CCGCTCGAGAGCTCTAGAAGCATAACTCCATAGC |  |
| CPD-F | GTTCTTATCCTGCTTCCATTTG | for RT-PCR |
| CPD-R | CGAATCACTCTTCATTGCCC |  |
| DWF4-F | CCACAACACTCGGTGACTTCA |  |
| DWF4-R | TCAGCTGATACGATCGTTGGTT |  |
| SlDWARF-F | AAAATTGATGAGTTTATGAGATCCC |  |
| SlDWARF-F | CAAGCATATCATGTTGAATTTCCT |  |
| SlCPD-F | CTTCTCTCCGAGCTGTTCATCTAG |  |
| SlCPD-R | GAAGGAAAACAGAGAGTTCCACTC |  |
| SlUBI3-F | GTGTGGGCTCACCTACGTTT |  |
| SlUBI3-R | ACAATCCCAAGGGTTGTCAC |  |
| Act2-F | AACTCTCCCGCTATGTATGTCG |  |
| Act2-R | GAGGTAATCAGTAAGGTCACGTCC |  |
| FRK1 qRT-F | AGCGGTCAGATTTCAACAGT | for qRT-PCR |
| FRK1 qRT-R | AAGACTATAAACATCACTCT |  |
| Actin2 qRT-F | TGTGCCAATCTACGAGGGTTT |  |
| Actin2 qRT-R | TTTCCCGCTCTGCTGTTGT |  |
| SlPR1b1 qRT-F | CTGGTGCTGGGGAGAATC |  |
| SlPR1b1 qRT-R | GTCCGATCCAGTTGCCTACA |  |
| SlPR2 qRT-F | TGTAGACATGACGTTGATTGGCA |  |
| SlPR2 qRTR-R | ACGGAAGTGAAATCTGTCCTGG |  |
| SlSERK3A qRT-F | TGTACACCGAAACCTACTTCGTTTA |  |
| SlSERK3A qRT-R | ACCAGTAGATAAATATTCAGGGGCA |  |
| SlSERK3B qRT-F | CCATGCTCACCATCCTCAC |  |
| SlSERK3B qRT-R | TGTGCTGCTGGAAGAATAGC |  |
| SlBIR3 qRT-F | AGTTTGGTTATGTGCCTCCTG |  |
| SlBIR3qRT-R | TACCTTTGAATCCCTCCTCC |  |
| SlUBI3-qRT-F | GTGTGGGCTCACCTACGTTT |  |
| SlUBI3-qRT-R | ACAATCCCAAGGGTTGTCAC |  |
